# Biobased Poly(dodecylene Furanoate) with Inherent Advantages in Performance and Circularity

**DOI:** 10.1002/cssc.202501080

**Published:** 2025-07-10

**Authors:** Hesham Aboukeila, Eswara Rao Chokkapu, Hang‐Fei Tu, Onkar Singh, Wilfred T. Diment, Shu Xu, Meltem Urgun‐Demirtas, John Klier, George W. Huber, Brian P. Grady, Eugene Y.‐X. Chen

**Affiliations:** ^1^ School of Sustainable Chemical, Biological and Materials Engineering University of Oklahoma Norman OK 73019 United States; ^2^ Department of Chemistry Colorado State University Fort Collins Colorado 80523‐1872 United States; ^3^ Department of Sustainable Materials and Processes Applied Materials Division Argonne National Laboratory Lemont IL 60439 United States; ^4^ Northwestern Argonne Institute of Science & Engineering Evanston IL 60208 United States; ^5^ Department of Chemical and Biological Engineering University of Wisconsin ‐ Madison 1415 Engineering Drive Madison Wisconsin 53706 United States; ^6^ Present address: State Key Laboratory of Coordination Chemistry, School of Chemistry and Chemical Engineering Nanjing University Nanjing 210023 P. R. China

**Keywords:** biobased polymer, biodegradation, chemical circularity, gas barrier, Poly(dodecylene 2,5‐furanoate) (PDDF)

## Abstract

Biobased polymers are gaining traction toward more sustainable flexible‐film packaging, yet overcoming trade‐offs between their performance properties and end‐of‐life (EoL) options still remains a challenge. Here, it is shown that biobased poly(dodecylene 2,5‐furanoate) (PDDF), synthesized via both step‐growth polycondensation and chain‐growth ring‐opening polymerization methods, exhibits advantages not only in gas barrier properties but also in EoL options due to its biodegradability and closed‐loop chemical circularity. Specifically, PDDF displays significantly lower oxygen and carbon dioxide permeability than commercial poly(butylene adipate*‐co*‐terephthalate) (PBAT) and linear low‐density polyethylene , alongside a markedly higher modulus (by ≈3 ×) and reduced water vapor transmission rate compared to PBAT. This superior performance is attributed to the inherently rigid, polar, H‐bonding furan rings that enhance chain interaction, packing and crystallinity and thus reduce free volume impeding gas diffusion, while the long hydrophobic dodecylene segments inhibit water permeation. Furthermore, PDDF can be recycled back to its cyclic monomer by base‐catalyzed depolymerization or diester and diol monomers by simple methanolysis. These superior barrier properties, coupled with biodegradation and closed‐loop circularity, highlight the potential of the biobased PDDF as a more sustainable alternative for packaging.

## Graphical abstract

Thanks to its inherent biomass furan motif that can promote polymer H‐bonding networks, biobased polyester PDDF is shown to exhibit advantages not only in performance (e.g., superior modulus, crystallinity, gas barrier properties) but also in end‐of‐life management (biodegradability and closed‐loop chemical circularity), as compared to petroleum‐based incumbent materials such as poly(butylene adipate*‐co*‐terephthalate).

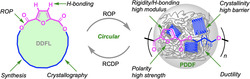

## 1 Introduction

The packaging industry is the largest consumer of plastics, driven by a significant shift from reusable containers to single‐use options, which has intensified waste management challenges.^[^ ^1^ ^]^ Most plastics, including the two largest volume plastics, polyethylene, and polypropylene, are fossil‐based and non‐biodegradable, leading to environmental accumulation and pollution.^[^ ^2^, ^3^ ^]^ Despite global efforts, only a small percentage of post‐consumer plastics is recycled, and recycling rates for materials used in flexible films such as low‐density polyethylene (LDPE) and linear low‐density polyethylene (LLDPE) remain particularly low (≈3.5%) due to reprocessing difficulties.^[^ ^4^ ^]^ As a result, leading organizations such as the United Nations,^[^ ^5^, ^6^ ^]^ the World Economic Forum,^[^ ^7^ ^]^ World Health Organization,^[^ ^8^ ^]^ and the European Union^[^ ^9^ ^]^ have prioritized plastic pollution mitigation. Yet, the plastics industry remains largely reliant on linear, non‐circular economy models.^[^ ^7^, ^10^ ^]^ With ≈400 million metric tons of plastic waste generated annually,^[^ ^4^ ^]^ nearly half of which is originated from packaging, there is an urgent demand for biodegradable and/or chemically circular alternatives to conventional films such as LDPE and LLDPE.

Biomass‐based, biodegradable, and circular polymers are key to the sustainable materials development.^[^ ^11^, ^12^, ^13^, ^14^ ^]^ Biodegradable polyesters such as polylactic acid (PLA),^[^ ^15^, ^16^, ^17^ ^]^ polyhydroxyalkanoate (PHA),^[^ ^18^, ^19^ ^]^ poly(butylene succinate) (PBS),^[^ ^20^, ^21^ ^]^ polyhydroxybutyrate (PHB),^[^ ^22^, ^23^ ^]^ and poly(butylene adipate*‐co*‐terephthalate) (PBAT)^[^ ^24^, ^25^ ^]^ show promise due to their biodegradability and adequate thermomechanical properties.^[^ ^26^ ^]^ Among them, fossil‐based, biodegradable PBAT and PBS emerged as more suitable alternatives for flexible packaging. Although biobased PBS is commercially available while biobased PBAT could be made commercially, high cost has significantly limited their adoption. Other biobased alternatives exhibit limitations to be overcome. For example, brittleness and high permeability (particularly toward water vapor) of PLA, poor processability of starch‐ and cellulose‐based materials,^[^ ^27^, ^28^ ^]^ and brittleness and poor impact resistance of PHB^[^ ^29^ ^]^ hinder broader adoption of such biobased materials.

Recent research focuses on improving performance of biobased plastics through innovative formulations. One attractive route is the use of 2,5‐furandicarboxylic acid (FDCA), a biomass‐derived monomer identified by the U.S. Department of Energy as a key replacement for petroleum‐based terephthalic acid^[^ ^30^ ^]^ in poly(ethylene terephthalate) (PET) production. FDCA is derived from diverse biomass sources such as sugars, starches, and lignocellulose, making it a versatile biobased precursor for polyester production.^[^ ^31^ ^]^ Relative to PET, FDCA‐derived poly(ethylene furanoate) (PEF)^[^ ^32^ ^]^ offers superior gas barrier properties, tensile strength, and higher glass‐transition temperature (*T*_g_), making it more ideal for long‐shelf‐life packaging. Companies such as Avantium are advancing large‐scale FDCA production from biomass.^[^ ^33^ ^]^ However, economic and recycling infrastructure challenges remain, along with processing limitations, due to PEF's thermal sensitivity. Beyond PEF, FDCA‐based polyesters, including poly(butylene adipate*‐co*‐furanoate), poly(propylene furanoate), and poly(butylene furanoate), further modulate thermomechanical performance and barrier properties.^[^ ^32^, ^33^ ^]^ Another promising component is 1,12‐dodecanediol (1,12‐DD), a long‐chain aliphatic diol from biomass, which can be synthesized through biomass routes through cellular biotransformation in *Escherichia coli*.^[^ ^34^ ^]^ Used in coatings, lubricants, and plastics, 1,12‐DD improves chemical resistance and reduces water absorption in polyesters and polyurethanes.^[^ ^32^, ^33^ ^]^ Its integration with FDCA enables the tailoring of polymer properties for specific applications.^[^ ^1^, ^35^ ^]^

The scalable production, end‐of‐life (EoL) management, and cost present three key barriers to biobased plastics adoption. In synthesis, the traditional step‐growth polycondensation (SGP) method involving multiple stages is commonly employed, but ring‐opening polymerization (ROP) of cyclic monomers or oligomers allows the synthesis of high‐molecular‐weight, low‐coloration polyesters more efficiently and rapidly. ROP proceeds via a chain‐growth mechanism with fast kinetics and requires no byproduct removal (unlike the SGP process), enabling the production of polymers with precise control over molecular weight by simply adjusting the monomer‐to‐initiator ratio. While recent studies show potential in synthesizing medium‐molecular‐weight furanic polyesters *via* ROP, challenges in reaching bottle‐grade molecular weight and scaling persist.^[^ ^32^, ^36^ ^]^ EoL management^[^ ^37^, ^38^ ^]^ is another key barrier to bioplastics adoption. Biodegradation and composting can convert post‐consumer polymers to CO_2_, H_2_O, and other harmless byproducts. In this context, furan‐based polyesters are susceptible to enzymatic degradation,^[^ ^39^, ^40^, ^41^ ^]^ while chemical recycling can offer closed‐loop circularity by recovering building‐block monomers for virgin‐quality plastic production, though infrastructure remains underdeveloped. The cost of biobased feedstocks is typically higher than fossil‐based ones, and the use of biobased plastics as direct replacements for incumbent plastics presents the third key barrier to bioplastics adoption. To address this challenge and compete at scale with fossil‐based polymers, new biobased polymers must demonstrate performance‐ and recyclability‐advantaged properties that harness the inherent chemical functionalities of the bio‐feedstocks.^[^ ^39^, ^40^, ^41^ ^]^

**Figure 1:**
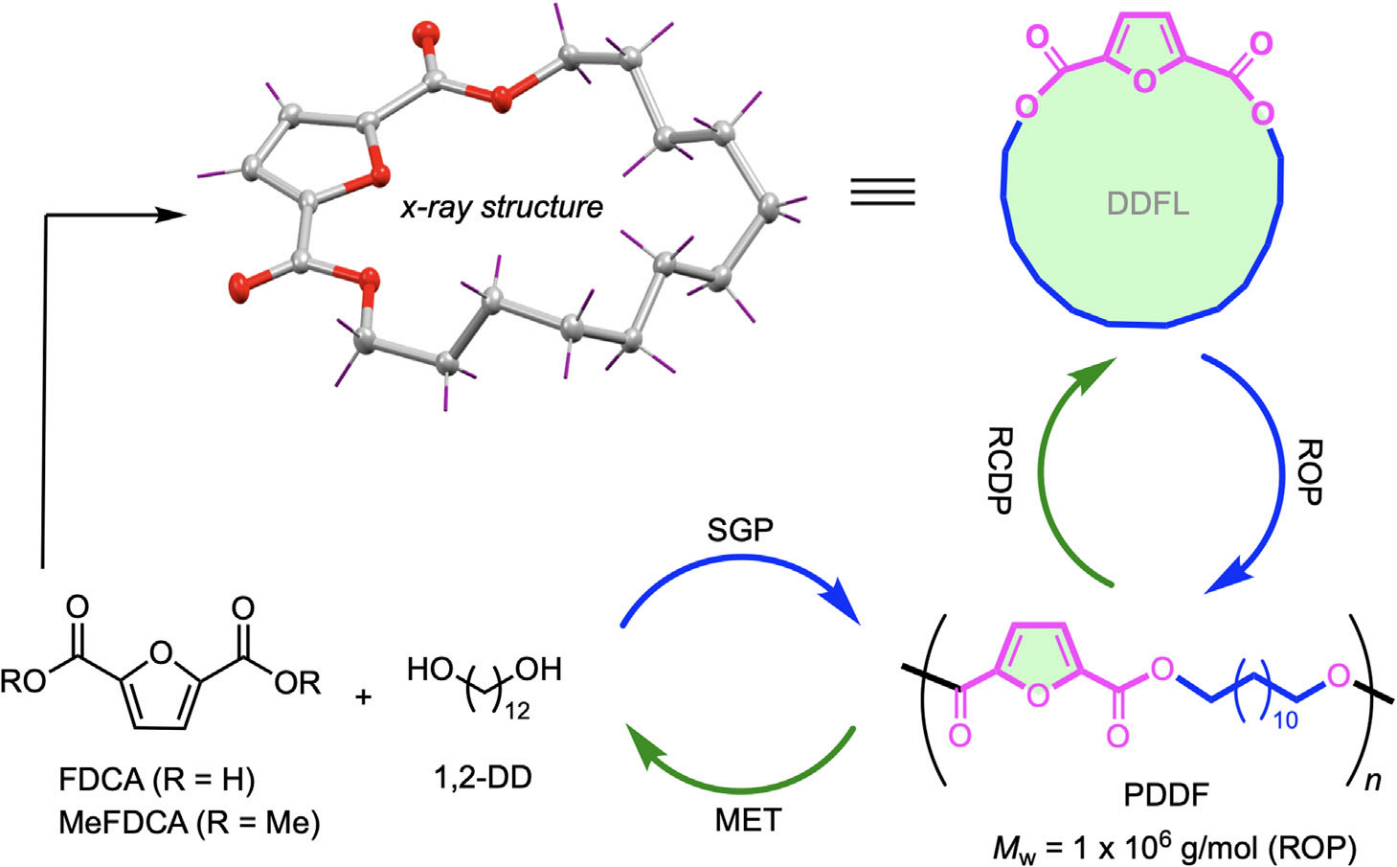
Synthesis and chemical circularity of PDDF through dual circular paths via ring‐opening polymerization (ROP)/ring‐closing depolymerization (RCDP) and step‐growth polymerization (SGP)/methanolysis (MET) routes.

In this study, we synthesized and characterized medium‐ to high‐molecular‐weight poly(dodecylene 2,5‐furanoate) (PDDF) *via* both SGP of FDCA with 1,12‐DD and ROP of the lactone monomer, dodecylene 2,5‐furanoate lactone (DDFL), the latter of which produced PDDF with weight‐average molecular weight (*M*_w_) up to 1.0 × 10^6^ g mol^−1^ (Da) (**Figure** **1**). Characterizations included monomer and polymer structures via single‐crystal X‐ray diffraction analysis and wide and small‐angle X‐ray scattering (WAXS/SAXS) studies; thermal, mechanical, and rheological properties; gas barrier performance; and biodegradation in fresh water, soil, and compost environments. A prior synthesis of PDDF *via* transesterification using 2,5‐dimethylfurandicarboxylate was reported by Papageorgiou *et al.* ^[^ ^1^, ^35^ ^]^ but ROP and SGP routes to high‐molecular‐weight PDDF have not been previously reported. Comprehensive properties of PDDF, including crystallization behavior, rheology, barrier, and biodegradability are reported for the first time. As comparative study, data on two commercial flexible packaging materials LLDPE and PBAT from our previous work are included.^[^ ^42^ ^]^ Notably, we demonstrate the chemical recyclability of PDDF to its monomers *via* catalyzed depolymerization to cyclic monomer DDFL for repolymerization via ROP and methanolysis to diester and diol monomers for repolymerization via SGP. This study underscores the potential of PDDF as a partially biodegradable, circular, and high‐performance biobased plastic for packaging applications.

## 2 Results and Discussion

### 2.1 Synthesis and Characterization of Cyclic Monomer DDFL for ROP

The synthesis of DDFL was initially accomplished through two approaches: 1) Yamaguchi esterification of FDCA with 1,12‐dodecanediol (1,12‐DD) (Scheme S1, Figure S1, Supporting Information), and 2) a two‐step process in which FDCA was first converted into 2,5‐furandicarbonyldichloride using thionyl chloride, followed by esterification with 1,12‐DD in the presence of Et_3_N to form a mixture of cyclic furanic oligoesters (Scheme S2 and S3, Figure S2 and S3, Supporting Information). Column chromatography (15% hexanes/EtOAc) was used to isolate pure DDFL in 19% yield. A more efficient strategy was later established via ring‐closing depolymerization (RCDP) of the SGP‐derived PDDF, affording DDFL in 71% yield. Structural identity and purity were confirmed by ^1^H and ^1^ ^3^C NMR (Figure S1–S3, Supporting Information). The comparative efficiency of these methods underscores the synthetic advantage of the RCDP route.

**Figure 2:**
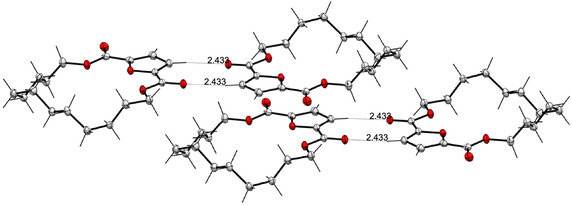
The molecular structure of the cyclic monomer DDFL obtained by single‐crystal X‐ray diffraction analysis and its expanded packing structure (*a* + 2, *b* + 1.5, *c* + 0). Carbon atoms in gray, oxygen atoms in red, and hydrogen atoms in black. The crystal data was deposited at the Cambridge Crystallographic Database Centre as CCDC‐2 359 161.

The monomeric nature and molecular structure of DDFL was further confirmed by single‐crystal X‐ray diffraction analysis (**Figure** **2**). DDFL crystallizes in a monoclinic system with unit cell parameters *a* = 9.4333 Å, *b* = 9.4417 Å, *c* = 11.1036 Å, and *α* = 85.420° *β* = 66.219°, *γ* = 74.516°. Notably, significant intermolecular interactions, including H‐bonding and van der Waals forces, were observed, contributing to the stability of the crystal structure. The expanded packing structure reveals close intermolecular interactions between the C–H hydrogen on the furan ring and the carbonyl oxygen of the ester, with a O–H distance of 2.433 Å and H–O–C angle of 143.38° (Figure 2). The p*K*_a_ of a phenyl hydrogen is 43–45, while the p*K*_a_ of a 2/5 (*ortho*)‐hydrogen in furan is 35.6 for significantly higher acidity (i.e., a better H‐bonding donor), owing to the inductive electron‐withdrawing effect of the oxygen on the furan ring, which stabilizes the resultant anion. Although the hydrogen atoms in DDFL are 3/4 (*meta*) rather than 2/5 (*ortho*), the presence of two electron‐withdrawing ester groups on both sides of the furan ring is expected to further assist in stabilizing the resultant anion. A p*K*_a_ of 27.5 was calculated for a 3‐H in a related furan heterocycle.^[^ ^43^ ^]^ Overall, these observations indicate significantly greater polarization of the C–H bond in furan rings over the equivalent in phenyl rings, suggesting that weak H‐bonding could occur,^[^ ^43^ ^]^ verified experimentally here.

Although these H‐bonding interactions observed in the cyclic DDFL monomer could vary in the corresponding linear polymer upon the ROP reaction, all structural motifs are identical, except for cyclic versus linear forms, which should render such interactions intact in the polymer. Hence, it can be expected the H‐bonding network would also exist in the polymer structure, which is a commonly practiced extrapolation made from monomer to polymer in the context of hydrogen bonds (as obtaining single‐crystal structures of synthetic polymers is not possible). Overall, the detailed analysis of the crystal structure of the monomer DDFL provided insights into the chain packing structure of the corresponding polymer PDDF and thus its suitability for packaging applications in the context of barrier performance, mechanical robustness, and thermal stability, all of which would be enhanced by the presence of a H‐bonding network structure present in the polymer material.

### 2.2 Synthesis of Medium‐ to High‐Molecular‐Weight PDDF by SGP and ROP

The ROP of DDFL was performed in solution or in melt, and selected results were summarized in **Table** **1**. Several molecular catalysts were screened, including a commercial lanthanum [La]‐based catalyst, La[N(SiMe_3_)_2_]_3_,^[^ ^44^ ^]^ and a commercial superbase catalyst, ^*t*^Bu‐P_4_ (1‐*tert*‐Butyl‐4,4,4‐tris(dimethylamino)‐2,2‐bis[tris(dimethylamino)‐phosphoranylidenamino]2*λ* ^5^,4*λ* ^5^catenadi(phosphazene)). At first, ^*t*^Bu‐P_4_ was shown to be an effective catalyst for the ROP of DDFL (Table 1, runs 1–3); however, attempts to produce high‐molecular‐weight PDDF with ^*t*^Bu‐P_4_ by employing a [DDFL]/[ ^*t*^Bu‐P_4_] ratio of 300:1 resulted in a low monomer conversion of only 19% (Table 1, run 4). On the contrary, the [La] complex, when combined with an initiating alcohol, PhCH_2_OH (BnOH), was shown to be highly effective for this ROP, producing PDDF with controlled molecular weight from low number‐average molecular weight (*M*_n_) = 18.3 kDa to high *M*_n_ = 764 kDa or weight‐average molecular weight (*M*_w_) = 1.0 MDa (Table 1, runs 5‐12). Several organic and metal‐based catalysts, including 1,8‐diazabicyclo[5.4.0]undec‐7‐ene and tin(II) 2‐ethylhexanoate (Sn(Oct)_2_), were also screened, but they were much less effective than the [La] catalyst. It should be pointed out here that the effectiveness of the ROP hinges on the availability of the high purity cyclic DDFL, which can be obtained in 71% yield from the catalyzed depolymerization of PDDF, providing a closed‐loop strategy for PDDF (*vide infra*).

**Table 1:** Selected results of the ROP of DDFL.

Run	catalyst	[M]/[Cat.]/[I]a)	Solvent	Temp. [°C]	Time [h]	Conv. [%]b)	*M*_n_ [kDa]c)	*Ð* c)
1	^*t*^Bu‐P_4_	100:1:1	neat	110	4.5	74	61.7	3.05
2	^*t*^Bu‐P_4_	100:1:1	THF	25	3	69	15.1	2.09
3	^*t*^Bu‐P_4_	100:1:1	THF	60	3	61	10.9	2.40
4	^*t*^Bu‐P_4_	300:1:1	THF	60	3	19	/	/
5	[La]	100:1:3	neat	110	4.5	100	31.2	1.36
6	[La]	100:1:3	DCM	25	0.5	100	25.6	1.47
7	[La]	100:1:3	DCM	25	3	100	25.0	2.30
8	[La]	100:1:3	DCM	60	3	100	18.3	2.43
9	[La]	1000:1:3	DCM	25	3	100	134	2.19
10	[La]	3300:1:3	DCM	25	1.5	97	227	1.60
11	[La]	6700:1:3	DCM	25	1.5	39	520	1.60
12	[La]	20 000:1:3	neat	125	14	38	764	1.31

a) Conditions: Monomer (M) (0.5 mmol); catalyst (cat.) = ^*t*^Bu‐P_4_, or [La] = La[N(SiMe_3_)_2_]_3_; initiator (I) = BnOH; solvent (0.2 mL), THF = tetrahydrofuran, DCM = dichloromethane, neat = no solvent.

b) Determined by ^1^ H NMR in CDCl_3_.

c) Number‐average molecular weight (*M*_n_) and dispersity index (*Ð* = *M*_w_ *M*_n_^−1^) determined via size exclusion chromatography (SEC) at 40 °C in CHCl_3_ coupled with a Wyatt DAWN HELEOS II multi (18)‐angle light scattering detector and a Wyatt Optilab TrEX dRI detector for absolute molecular weight.

The traditional diacid (A_2_) and diol (B_2_) SGP route was also employed for the synthesis of PDDF (PDDF‐SGP) with a medium molecular weight, which was performed through a two‐step procedure, esterification followed by polycondensation. In the first step, 1,12‐DD was melted by slowly increasing the temperature to 100 °C under a slow nitrogen flow and continuous agitation. FDCA was then added to the molten DD, and the temperature gradually increased to 170°C. At this temperature, titanium isopropoxide (0.1 mol % of acid) was introduced and the temperature was further raised to 200°C at a controlled rate of 5°C/30 min. The esterification reaction was allowed to proceed for the next 24 h. Following the esterification reaction, in the second step another portion of titanium isopropoxide (0.1 mol % of acid) was added and the vacuum was reduced to less than 0.1 mbar, initiating the polycondensation phase. The temperature then increased to 230°C and polycondensation continued until a Weissenberg effect was observed. The average molecular weight and dispersity (*Ð*) of PDDF‐SGP are compared to commercial PBAT in **Table** **2**.

**Table 2:** Molecular weights for PBAT and PDDF prepared by SGP and ROP.

Sample	*M_n_* kDa	*M_w_* kDa	Ð*M*_w_ *M*_n_^−1^
PBATa)	59.9	97.9	1.60
PDDF‐SGPa)	31.6	52.6	1.70
PDDF‐ROPb)	227	363	1.60

a) Relative to polystyrene standards.

b) From run 10, Table 1, absolute molecular weight.

### 2.3 Thermal Stability, Crystallization, and Melting Behaviors

**Figure 3:**
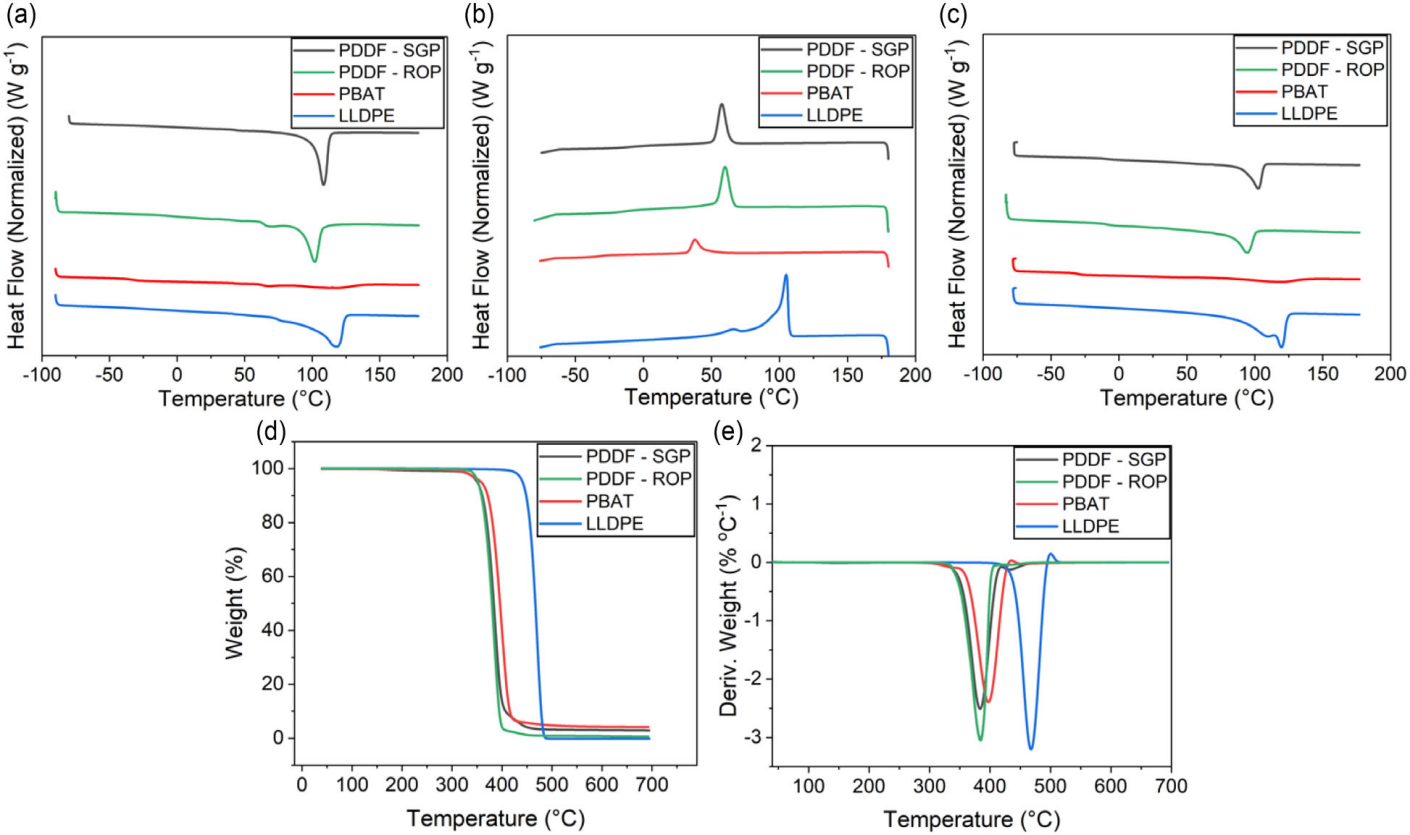
Thermal properties of PDDF, compared to commercial materials PBAT and LLDE. a) DSC first heating cycle. b) DSC first cooling cycle. c) DSC second heating cycle. d) TGA thermogram overlays. e) Derivative TGA (DTGA) thermogram overlays.

**Figure** **3** and **Table** **3** show the temperatures for different decomposition stages, including the temperature at which 5% decomposition occurs (*T*_d,5%_), the temperature of maximum decomposition rate (*T*_d,max_), and the residual mass at 600 °C (R_600°C_) obtained from thermogravimetric analysis (TGA). PBAT exhibits similar thermal stability to PDDF but with slightly higher *T*_d,5%_ (by ≈6 °C) and *T*_d,max_ (by ≈10 °C) values, likely due to the slightly higher stability of the phenyl ring versus the furan ring (Figure 3d,e). PDDF polyesters have a lower residue at 600 °C compared to PBAT, again attributed to the presence of the furan ring in PDDF versus the phenyl ring in PBAT. Additionally, PDDF‐ROP showed a lower residue at 600 °C (0.8%) compared to PDDF‐SGP (3.1%). One possible explanation is that the ROP of the cyclic monomer was more controlled and performed under relatively milder conditions (25 to 125 °C), thus eliminating or suppressing possible reaction by‐products occurred under the harsh SGP conditions (230 °C, vacuum).

**Table 3:** Thermal properties measured by DSC, TGA, and DMA.

Sample	First Heating		First Cooling		Second Heating			DMA	TGA		
	$T_{m}$ °C	$\Delta H_{m}$ *J* *g* ^−1^	$T_{c}$ °C	$\Delta H_{c}$ *J* *g* ^−1^	Tg°C	Tm°C	$\Delta H_{m}$ *J* *g* ^−1^	Tg°C	$T_{d,5\%}$ °C	$T_{\text{d,max}}$ °C	R_600 °C_%
LLDPE	116	131	106	109	−	116	127	−	441	453	0
PBAT	115	34	38	20	−30	118	20	−27	358	376	4.3
PDDF‐SGP	108	80	58	63	−9	102	52	−6	351	366	3.1
PDDF‐ROP	102	78	60	52	−10	95	61	2	352	363	0.8

Crystallization and melting behaviors of PDDF polyesters were investigated using differential scanning calorimetry (DSC), as shown in Figure 3, with the resultant melting temperature (*T*_m_), enthalpy of melting (Δ*H*_m_), crystallization temperature (*T*_c_), enthalpy of crystallization (Δ*H*_c_), and *T*_g_ summarized in Table 3. Upon heating, PDDF polyesters presented a well‐defined melting peak, indictive of their semi‐crystalline nature. A sharp endothermic melting peak was observed for PDDF and LLDPE during both the first and second heating cycles, whereas a broader peak was noted for PBAT (Figure 3a,c). PDDF polyesters exhibited a peak at higher temperature during cooling versus PBAT, indicative of a faster crystallization rate (Figure 3b), likely due to the longer methylene sequence in the PDDF chain. High‐molecular‐weight PDDF‐ROP (*M*_n_ = 227 kDa) displayed a lower *T*_m_ (by ≈7 °C) and similar *T*_g_ compared to medium‐molecular‐weight PDDF‐SGP (*M*_n_ = 31.6 kDa). A lower *T*_m_ indicates thinner crystals, an indication that was confirmed by SAXS measurements (*vide infra*). Notably, PDDF polyesters showed a significantly higher Δ*H*_m_ (by ≈3 ×) compared to PBAT, presumably due to the presence of the longer alkyl chain in PDDF, suggestive of a higher degree of fractional crystallinity that was confirmed by WAXS measurements (*vide infra*). Owing to differences in molecular weights and DSC conditions, the *T*_m_ and *T*_g_ values of the current PDDF materials differed somewhat from those reported in literature (*T*_m_ ≈ 110 °C, *T*_g_ ≈ −22 °C).^[^ ^1^, ^45^ ^]^

**Figure 4:**
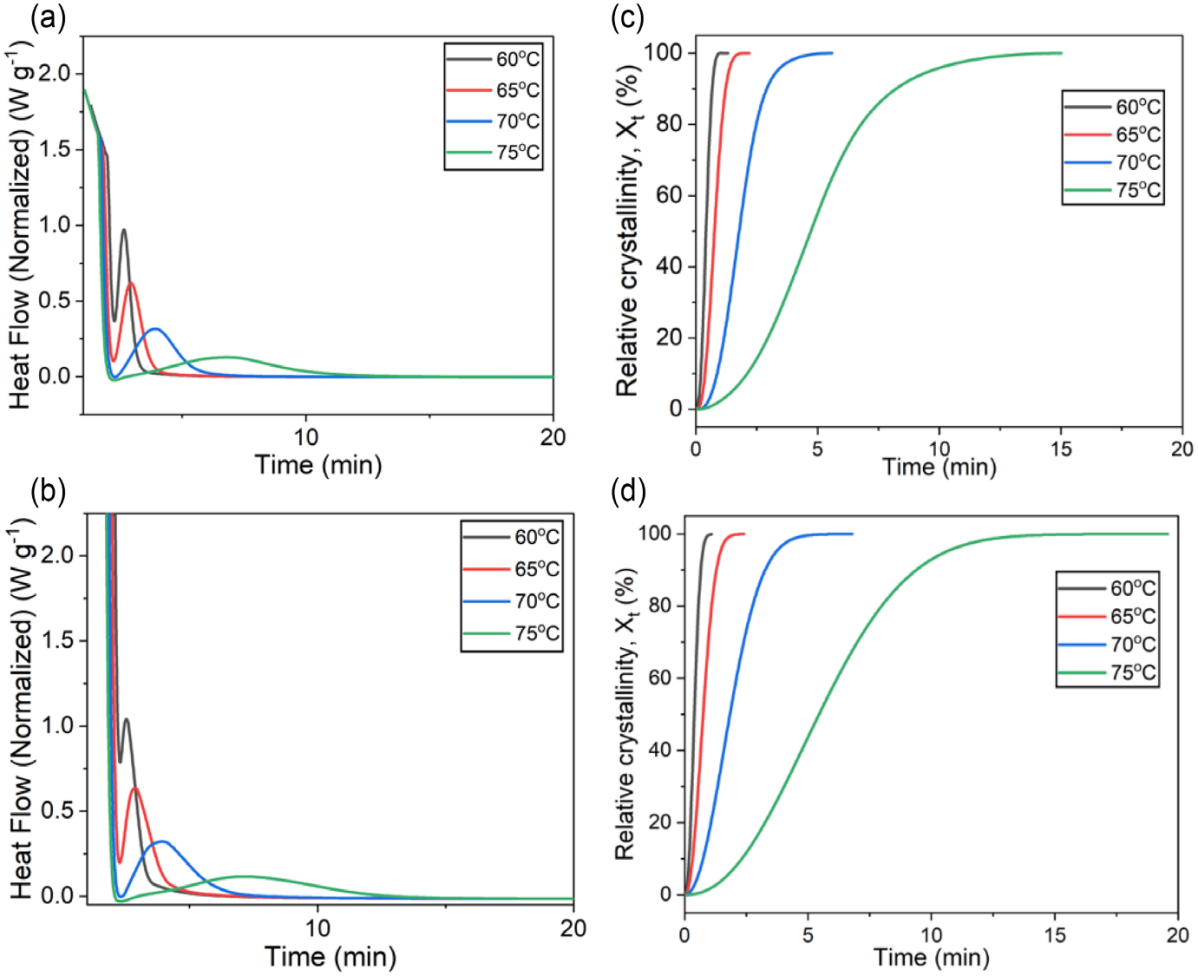
Isothermal crystallization kinetics. a) Isothermal crystallization for PDDF‐SGP. b) Avrami model results for PDDF‐SGP. c) Isothermal crystallization for PDDF‐ROP. d) Avrami model results for PDDF‐ROP.

Isothermal crystallization kinetics of PDDF polyesters were studied at different temperatures using DSC with results reported in **Figure** **4**. The well‐known Avrami model was utilized to gain insights into the isothermal crystallization process. The Avrami model illustrates the time‐dependent relative crystallinity (*X*_*t*_) at a constant temperature, as presented in Equation (1)

(1) $$X_{t}=1-\exp\left(-kt^{n}\right)$$

The half‐time for 100% relative crystallization (*t*_1/2_) was calculated using Equation (2)

(2) $$t_{\frac{1}{2}}={\left(\frac{\ln2}{k}\right)}^{\frac{1}{n}}$$

where *k* is the rate constant for crystallization that is a function of nucleation and growth rates, and *n* is the Avrami exponent. The crystallization half‐time (*t*_1/2_) and the Avrami model parameter are reported in **Table** **4**. The rate of crystallization decreases with the increase in crystallization temperature, with no substantial difference between the two PDDF materials of different synthetic origins (SGP vs ROP), although there is a significant difference in molecular weight. The lack of difference in rate here is also found in the non‐isothermal crystallization results presented in Table 3. The Avrami exponent for all temperatures is between 2 and 3 but decreases as temperature increases. The growth mechanism changes from one that is equally distributed between two and one‐dimensional growth to one that is more one‐dimensional growth.^[^ ^46^ ^]^

**Table 4:** Avrami model parameters for PDDF.

Sample	Tc °C	t_1/2_min	kmin^−*n*^	n
PDDF‐SGP	60	0.42	6.42	2.54
	65	0.77	1.39	2.58
	70	1.78	0.16	2.50
	75	4.72	0.02	2.25
PDDF‐ROP	60	0.39	6.27	2.35
	65	0.75	1.32	2.21
	70	1.86	0.19	2.09
	75	5.01	0.02	2.20

### 2.4 Characteristics of PDDF Crystallites

**Figure 5:**
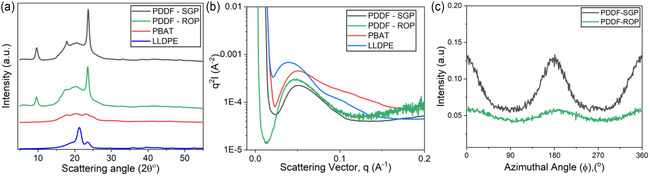
Properties of PDDF crystallites, compared to PBAT and LLDPE. a) WAXS profiles for PDDF, PBAT, and LLDPE. b) SAXS profiles for PDDF, PBAT, and LLDPE. c) Orientation from SAXS for PDDF‐SGP and PDDF‐ROP in the thickness (0° and 180°) direction.

**Figure** **5** illustrates the WAXS patterns of the PDDF polyesters, compared to PBAT and LLDPE, while **Table** **5** provides percentage crystallinity values for these polymers. For PBAT, scattering angles (2*θ*°) observed are 16.2°, 17.3°, 20.4°, 23.2°, and 24.8° corresponding to the planes (011), (010), (102), (100), and (111) respectively, consistent with the literature. For LLDPE, two sharp crystalline peaks can be observed at the 2 θ° values of 21.4° and 23.6° which are assigned to (110) and (200) planes. For PDDF, 2*θ*° are 9.8°, 17.9°, 21.6°, and 23.8° with the latter two scattering angles at approximately at the same angles as the planes (110), and (200) of LLDPE. The percentage crystallinity of PDDF (30.2%) is a bit more than half the percent crystallinity of LLDPE (50.4%); the ratio of these two values is roughly consistent with the Δ*H*_*m*_ values (Table 3). Moreover, PDDF has a higher percent crystallinity than PBAT (19.4%), which is due to the presence of a higher number of methylene units in PDDF leading to a more crystalline polyester. The increase in the molecular weight of PDDF‐ROP compared to PDDF‐SGP resulted in a decrease in percent crystallinity. The ratio of the peak heights for PDDF‐ROP and PDDF‐SGP observed in DSC analysis for the first heating, which also had room temperature annealing and hence should approximately correspond to the sample measured in WAXS, aligns (0.7) closely with the ratio of percent crystallinity for PDDF‐ROP and PDDF‐SGP derived from WAXS measurements (0.76).

**Table 5:** Crystallinity properties for PDDF polyesters, compared to PBAT and LLDPE.

Sample	WAXS	SAXS		
	$\chi_{c}\%$	q_max_ ${Å}^{-1}$	d‐SpacingÅ	Lamellar ThicknessÅ
LLDPE	50.4	0.039	160	80.8
PBAT	19.4	0.051	124	24.2
PDDF‐SGP	30.2	0.051	124	37.5
PDDF‐ROP	23.1	0.049	128	29.6

Figure 5 and Table 5 also summarized SAXS results for PDDF, PBAT, and LLDPE. As the crystallites are anticipated to have a lamellar shape, the peak observed in q^2^I(q) versus q represents the characteristic long spacing derived from Bragg's law. The long period (*d*‐spacing) is calculated using Equation (3) where *q*_max_ is the value where q^2^I(q) versus q.

(3) $$d=\frac{2\pi }{q_{\text{max}}}$$

The values of lamellar thickness reported in Table 5 are derived from the multiplication of fractional crystallinity from WAXS by the d‐spacing, that is, the difference in density between the two phases is ignored. The *d*‐spacing between PBAT and PDDF is the same so the lamellar thickness of PBAT is smaller. As mentioned earlier, the lamellar thickness for the PDDF‐SGP is larger than that for the PDDF‐ROP, consistent with DSC *T*_m_ data. In addition, the broadness of the SAXS peak for the PBAT is larger, consistent with the much broader peak in DSC heating scans. Consistent with the higher percentage crystallinity, the lamellar thickness for LLDPE is much larger.

To observe the change in orientation between the PDDF‐SGP and PDDF‐ROP in the thickness direction, 0° and 180°, samples were cut so that the beam was perpendicular to the thickness direction. SAXS patterns were examined for change in the transmissional intensities as a function of the azimuthal angle and the results are shown in Figure 5c. The scattering vector range used was the width of the scattering peak, that is, 0.03 and 0.1 A^−1^ for PDDF‐SGP and 0.02 and 0.1 A^−1^ for PDDF‐ROP. The lamellar thickness direction is aligned in the same direction as the thickness direction for both, with much higher alignment for the PDDF‐SGP. Presumably, the much higher molecular weight of PDDF‐ROP led to a higher number of entanglements, which restricts the chain mobility needed for lamellar alignment.

### 2.5 Mechanical and Rheological Properties

**Figure 6:**
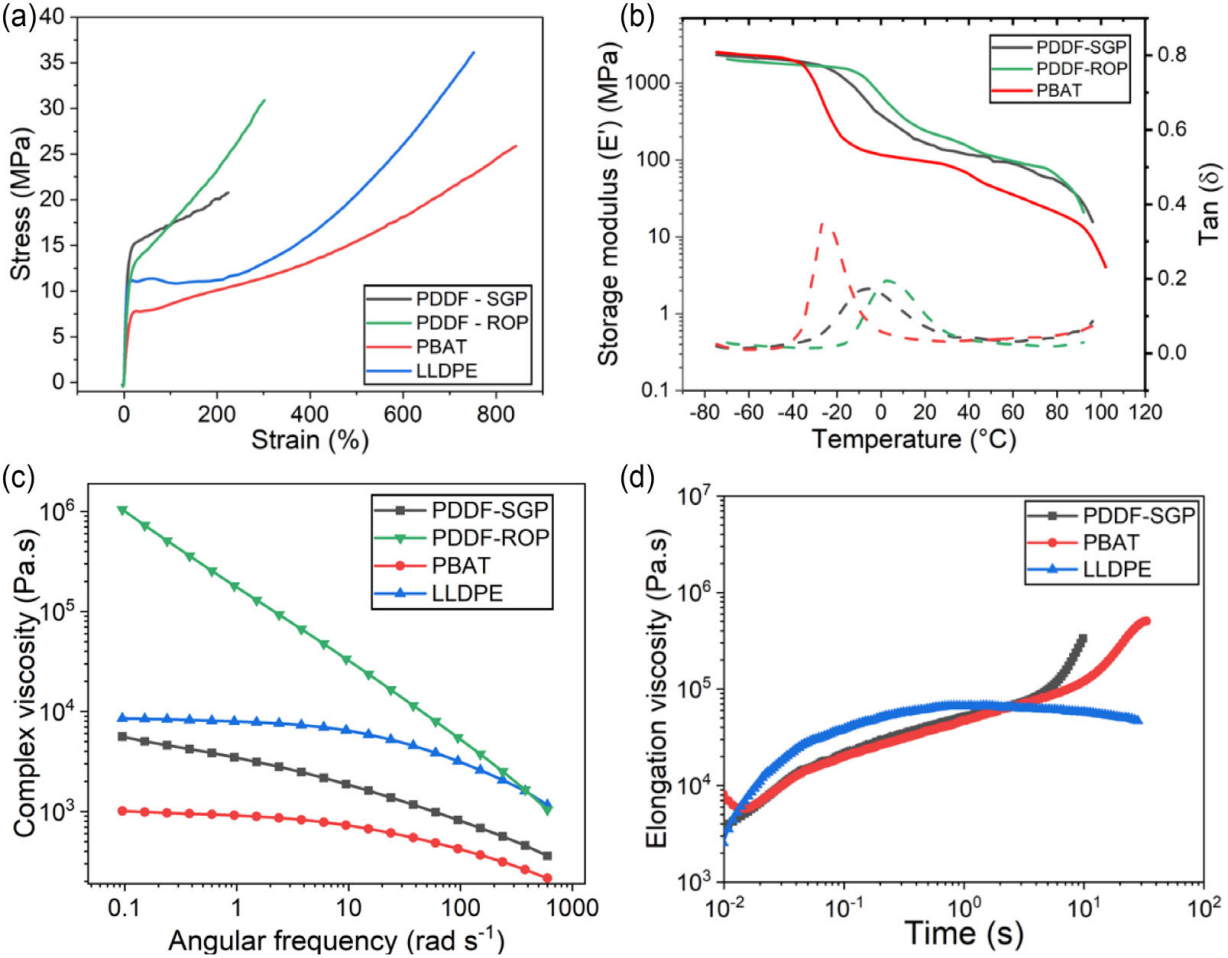
Mechanical, dynamic mechanical, and rheological properties of PDDF, compared to PBAT and LLDPE. a) Stress–strain curves for PDDF, PBAT, and LLDPE. b) DMA results for PDDF and PBAT. Storage modulus is represented as (‐ solid lines), and damping factor is represented as (‐ ‐ dashed lines). c) Complex viscosity curve of PDDF, PBAT, and LLDPE. d) Elongation viscosity curves for PDDF, PBAT, and LLDPE.

Stress–strain curves for PDDF‐SGP and PDDF‐ROP, compared to PBAT and LLDPE, are overlaid in **Figure** **6**a. Young's modulus (*E*), yield stress ($\delta_{y}$), yield strain ($\varepsilon_{y}$), stress at break ($\delta_{b}$), and elongation at break ($\varepsilon_{b}$) are calculated and reported in **Table** **6**. All four polymers exhibit ductile behavior with a high elongation at break. The measured *E*, $\delta_{y}$, $\delta_{b}$, and $\varepsilon_{b}$ values for LLDPE and PBAT agree with those reported in literature.^[^ ^47^, ^48^ ^]^

**Table 6:** Mechanical properties of PDDF, compared to PBAT and LLDPE.

Sample	EMPa	$\delta_{y}$ MPa	$\varepsilon_{y}$ %	$\delta_{b}$ MPa	$\varepsilon_{b}$%
LLDPE	255 ± 33	11 ± 0.3	19 ± 21	37 ± 4	645 ± 71
PBAT	78 ± 10	–	–	26 ± 3	815 ± 67
PDDF‐SGP	247 ± 7	–	–	21 ± 2	220 ± 32
PDDF‐ROP	165 ± 7	–	–	37 ± 3	280 ± 60
PDDF^[^ ^1^ ^]^	181 ± 16	9.5 ± 1.1	–	11 ± 0.9	130 ± 10

PDDF demonstrates outstanding mechanical performance, with Young's modulus *E* = 247 ± 7 MPa for the PDDF‐SGP and *E* = 165 ± 7 MPa for the PDDF‐ROP; both are significantly higher than PBAT's 78 ± 10 MPa, while the former is comparable to that of LLDPE (*E* = 255 ± 33 MPa). The higher modulus is a result of the higher crystallinity of the PDDF‐SGP. PDDF‐ROP exhibited a higher stress at break ($\delta_{b}$= 37 ± 3 MPa) and elongation at break ($\varepsilon_{b}$= 280 ± 60%) compared to PDDF‐SGP ($\delta_{b}$= 21 ± 2 MPa, $\varepsilon_{b}$= 220 ± 32%). As expected, PDDF is stiffer and has a lower elongation at break than PBAT due to PDDF's higher fractional crystallinity. Moreover, the mechanical properties of PDDF in this study significantly differ from those reported by Papageorgiou *et al.*,^[^ ^1^ ^]^ denoted as PDDF^[^ ^1^ ^]^ in Table 6 were showed much lower $\delta_{b}$ and $\varepsilon_{b}$ values, likely due to variations in molecular weights, synthesis processes, such as the completeness of transesterification, or differences in cooling methods after compression molding.

Figure 6b presents the storage modulus (*E'*) and damping factor (tan *δ*) as a function of temperature, measured by dynamic mechanical analysis (DMA). The DMA *T*_g_ was determined by the temperature that corresponded to the peak in tan *δ* curve. This *T*_g_ value was consistent with that obtained from DSC for PDDF‐SGP and PBAT; however, for PDDF‐ROP, the *T*_g_ obtained from DMA was higher (by 12 C°) than the *T*_g_ obtained from DSC as shown in Table 3. This difference is likely related to the much higher number of entanglements in the glass which shifts a mechanical *T*_g_ to a higher temperature.

Complex shear viscosities versus angular frequency at 190 °C for PDDF polyesters, compared to LLDPE and PBAT, are plotted in Figure 6c. Shear‐thinning behavior at high frequencies and a Newtonian plateau at low frequencies can be observed for PDDF‐SGP, LLDPE, and PBAT. The higher melt viscosity of PDDF‐SGP versus PBAT is somewhat surprising, given that the molecular weight of the former is ≈1/2 the molecular weight of the latter. A possible explanation is the long chain of the aliphatic diol causes the critical entanglement molecular weight of PDDF to be less than PBAT, while the other is that the friction coefficient is higher for the former. The very long chain of PDDF‐ROP showed very high melt viscosity due to its very high molecular weight, as well as a much lower frequency for the transition to the zero‐shear viscosity region. PDDF‐ROP showed a crossover viscosity at high frequencies with LLDPE and likely would have shown a crossover with PDDF‐SGP at a few thousand radians/sec. The shear rate within the extruder during film blowing is expected to be 45 s^−1^,^[^ ^49^ ^]^ which corresponds to a frequency of 283 radians/sec on Figure 6c. Hence, if the Cox–Merz rule is applicable to these polymers, then the extrusion energy required for PDDF‐ROP is about the same as that needed for LLDPE while for PDDF‐SGP the extrusion energy required is significantly lower. The low frequency tested limit was quite high because of thermal degradation of FDCA.^[^ ^50^, ^51^ ^]^

The five‐parameter Carreau–Yasuda was used to model the complex viscosities. The model is shown in Equation (4) and the results of which are reported in **Table** **7**.

(4) $$\frac{\eta \left(\omega \right)-\eta_{\infty }}{\eta_{0}-\eta_{\infty }}={\left[1+{\left(\omega \lambda \right)}^{a}\right]}^{\frac{n-1}{a}}$$

where $\eta_{\infty }$ is the infinite shear viscosity in (Pa.s), $\eta_{0}$ is the zero‐shear viscosity in (Pa.s), *ω* is the angular frequency in (rad s^−1^), *λ* is the characteristic relaxation time in (s), *a* is the transition parameter, and *n* is the flow index in Carreau–Yasuda model. The $\eta_{\infty }$ for the polymer melt is assumed to be zero. Fitted data showed good agreement with the experimental results; although the lack of a turnover frequency for PDDF‐ROP means that η_o_, *a*, and λ are not very accurate for this polymer. PDDF‐SGP had a lower flow index and a higher characteristic relaxation time than PBAT, reflective of a decrease in shear‐thinning behavior and a narrower Newtonian plateau region, respectively.

**Table 7:** Rheological properties of PDDF, compared to LLDPE and PBAT.

Sample	Shear viscosity				Elongation viscosity at Hencky strain of 2.5 and 0.3 s^−1^
	$\eta_{o}$ Pa.s	*n*	*a*	*λ* s	$\eta_{E}$ Pa.s
LLDPE	8711	0.12	0.62	0.01	47 000
PBAT	1006	0.67	0.76	0.12	350 000
PDDF ‐ SGP	9168	0.45	0.29	0.28	225 000
PDDF ‐ ROP	61 741 097	0.17	0.82	1354	–

Elongation viscosities versus time for PDDF polyesters, LLDPE, and PBAT at 130 °C and 0.3  $s^{-1}$ are plotted in Figure 6d. A lower temperature was chosen compared to the temperature used for shear viscosity measurements, as high temperatures lead to sample sagging. In addition, a Hencky strain of 2.5 at an elongational rate of 0.3 s^−1^ was found to be representative values for film blowing^[^ ^49^ ^]^ and these values are listed in Table 7. High elongation viscosity serves as an indicator of elevated melt strength, consequently contributing to a more stable bubble during the film‐blowing process. Strain hardening is characterized by an increase in elongation viscosity above the linear viscoelastic plateau.^[^ ^52^ ^]^ In this context, PDDF‐SGP and PBAT exhibit strain hardening, while the absence of strain hardening in LLDPE is attributed to the presence of short branches that allow the polymer chains to stretch without any entanglements. Strain hardening has been attributed to certain long chains remaining stretched until the sample's failure.^[^ ^53^ ^]^ Micic *et al.* reported that strain hardening is linked to improved stability of the bubble during the film‐blowing process.^[^ ^54^ ^]^ The very high elongation viscosity of PDDF‐ROP prevented the accurate measurement of elongation viscosity at 130 °C. The sample continued to slip because adequate adhesion to the grip requires a slight melting of the material. In other words, 130 °C was insufficient to lower the viscosity of the PDDF‐ROP sample to a level compatible with the equipment limits.

### 2.6 Gas Barrier Properties

**Figure 7:**
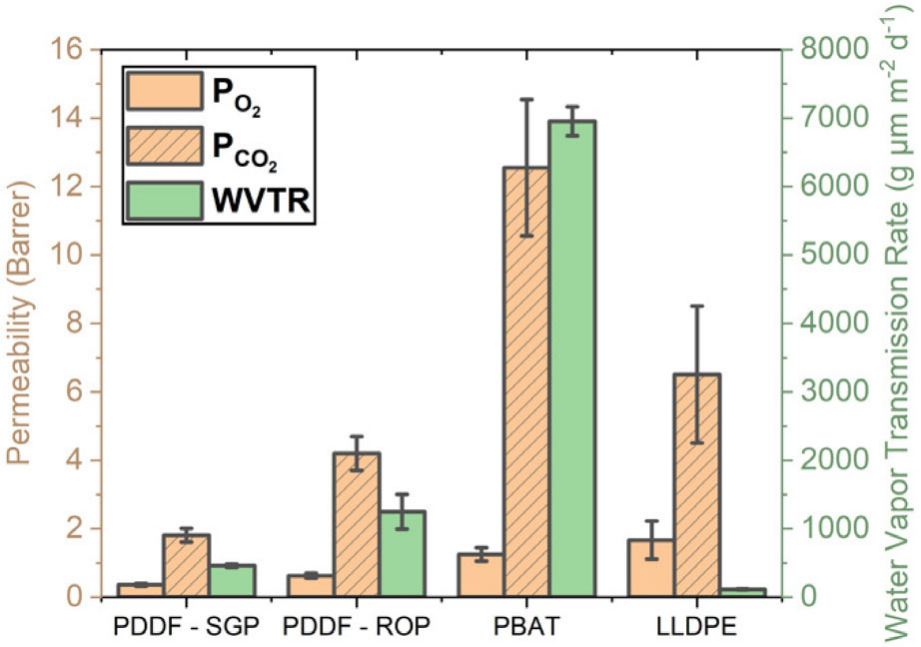
Gas barrier properties. Measurements of H_2_O, O_2_, and CO_2_ permeability of PDDF, compared to commercial packaging/barrier materials PBAT and LLDPE.

Oxygen (${\text{Ρ}}_{O_{2}}$) and carbon dioxide (${\text{Ρ}}_{CO_{2}}$) permeabilities are shown in **Figure** **7** with barrier improvement factor reported in **Table** **8**. ${\text{Ρ}}_{O_{2}}$ and ${\text{Ρ}}_{CO_{2}}$ for PBAT and LLDPE agree with the literature.^[^ ^55^, ^56^ ^]^ An increase in percent crystallinity leads to a decrease in gas permeability since permeability occurs almost exclusively through the amorphous regions. As expected, PDDF polyesters have a lower ${\text{Ρ}}_{O_{2}}$ and ${\text{Ρ}}_{CO_{2}}$ than PBAT due to the higher percent crystallinity of PDDF compared to PBAT.^[^ ^57^, ^58^ ^]^ Moreover, polyesters tend to have a lower ${\text{Ρ}}_{O_{2}}$ than polyolefins due to the presence of carbonyl and ester bonds, which led to a more hydrophilic structure and thus a lower ${\text{Ρ}}_{O_{2}}$ ^[^ ^57^ ^]^ PDDF‐ROP has a higher ${\text{Ρ}}_{O_{2}}$ and ${\text{Ρ}}_{CO_{2}}$ compared to PDDF‐SGP, and the higher percent of crystallinity of PDDF‐SGP is consistent with this observation as well as the lamellar orientation being higher for the PDDF‐SGP. PDDF polyesters and PBAT have a lower ${\text{Ρ}}_{O_{2}}$ than LLDPE making them good candidates for food packaging.

**Table 8:** Barrier improvement values for PDDF compared to LLDPE.

Sample	Oxygen Permeability	Carbon dioxide Permeability	Water vapor Transmission rate
LLDPE	1	1	1
PDDF‐SGP	4.6	3.6	0.2
PDDF‐ROP	2.7	1.6	0.1

Water vapor transmission rate (WTVR) in g μm *m* ^−2^ d^−1^ results for PDDF, LLDPE, and PBAT are also shown in Figure 7. PBAT has a much higher water vapor transmission rate (WVTR) than LLDPE and PDDF due to the hydrophilic nature of the polar functional groups such as hydroxyl, ester, and carboxyl groups, which form hydrogen bonds with water vapor molecules.^[^ ^59^ ^]^ Furthermore, an increase in percent crystallinity leads to a decrease in WVTR due to the increase in rigidity and decrease in allowed space for water vapor. PDDF‐SGP having a lower WVTR than PDDF‐ROP, consistent with the CO_2_ and O_2_ permeabilities. Overall, PDDF exhibits a significantly lower WVTR and ${\text{Ρ}}_{CO_{2}}$ , as well as a lower ${\text{Ρ}}_{O_{2}}$ than PBAT. Relative to LLDPE, PDDF shows much lower CO_2_ and O_2_ permeabilities, demonstrating performance advantages of PDDF over these two incumbent packaging/barrier materials.

### 2.7 Chemical Circularity and Biodegradation of PDDF

ROP produces condensation‐type polyesters through a chain‐growth mechanism, consisting of initiation, propagation, and termination steps. Without termination, polymers can reach an equilibrium with monomers, known as reversible polymerization. For propagation, both enthalpy (Δ*H*_p_) and entropy (Δ*S*_p_) of polymerization are typically negative, meaning polymerization occurs at low temperatures, below its ceiling temperature (*T*_ce_). Conversely, depolymerization occurs at high temperatures, above *T*_ce_. For pure monomers, *T*_ce_ = Δ*H*_p_Δ*S*_p_, but in solution, solvents increase monomer's entropy, lowering *T*_c_ or raising the floor temperature (*T*_fl_), as described by the Dainton and Ivin Equation (5).^[^ ^60^, ^61^ ^]^

(5) $$T_{\text{ce}}\left(or\right)T_{\text{fl}}=\frac{\Delta H{{}^{\circ}}_{P}}{\Delta S{{}^{\circ}}_{P}+R\ln{\left[M\right]}_{0}}$$

In ROP, small‐ to medium‐sized strained rings result in negative Δ*H*_p_ and Δ*S*_p_, where polymerization is driven by enthalpy. Depolymerization can be facilitated by high dilution or reactive distillation; the former shifts the equilibrium position toward the monomer state, favored by their higher translational entropy, whilst the latter ensures equilibrium is never established via constant monomer removal. For macro‐lactones (ring size >12 atoms) with non‐ or negligible ring strain, polymerization is driven by entropy (i.e., the gain in conformational entropy upon ring‐opening can effectively offset the loss in translational entropy due to polymerization) and thus regulated by *T*_fl_: high temperatures favor polymer formation and low temperatures favor monomer formation (the case for DDFL, a 19‐membered lactone).

**Figure 8:**
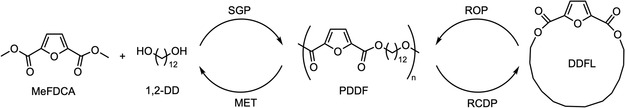
Dual closed circular chemical loops established by catalyzed ring‐closing depolymerization (RCDP)/repolymerization (ROP) and methanolysis (MET)/repolymerization (SGP).

**Figure 9:**
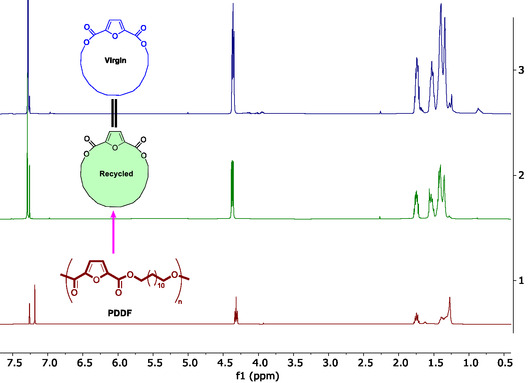
Chemical recyclability demonstration for PDDFL. ^1^H NMR spectra (CDCl_3_) of starting virgin monomer DDFL (top), recycled DDFL (second from top) monomer, and PDDF polymer by ROP (bottom).

Guided by the above thermodynamic principles, depolymerization of a ROP‐derived PDDF (*M*_n_ = 50.8 kDa, *Ð* = 1.84) film‐grade sample was first performed at room temperature (RT), considering the floor temperature phenomenon of this macro‐lactone monomer. Although the polymer indeed depolymerized to the cyclic monomer at RT in the presence of strong bases such as ^*t*^BuOK and ^*t*^Bu‐P_4_, the conversion was low when the equilibrium between the polymer and monomer was reached. Lewis acid catalysts such as phosphomolybdic acid and ZnCl_2_ were ineffective, recovering products that are mostly oligomers mixed with the olefinic side products caused by decomposition. To drive the reaction forward, a sublimation apparatus was employed, achieving ≈40% yield of the desired cyclic monomer (DDFL) with ^*t*^BuOK at the catalyst at 120 °C. Notably, polymer decomposition was observed at both RT and higher temperatures with ^*t*^BuOK. Switching to organic superbase ^*t*^Bu‐P_4_ as the catalyst, depolymerization at 120 °C afforded 71% of the pure DDFL (**Figure** **8**). Additionally, the recovered monomer DDFL can be fully repolymerized to PDDF, demonstrating the closed‐loop circularity of this biobased material (**Figure** **9**). Through consecutive polymerization‐depolymerization cycles, a circular cyclic monomer, liner polymer, cyclic monomer loop was achieved.

A second pathway to establish closed‐loop chemical recycling is through methanolysis of PDDF to the corresponding diester (A_2_) and diol (B_2_) monomers for repolymerization by SGP. Thus, the diester, dimethyl furan‐2,5‐dicarboxylate (MeFDCA), and the diol, 1,12‐DD, were recovered in quantitative yields using 30 wt% Et_3_N at 120 °C for 18 h. The recovered diester and diol were used for repolymerization via the SGP protocol without additional purification (Supplementary Figure S6, Supporting Information). These results demonstrate that the PDDF materials obtained by both ROP and SGP routes can be readily depolymerized under relatively mild conditions (120 °C) into either ROP or SGP starting monomers in high to quantitative yields, showing the potential for PDDF applied in a circular economy.

**Figure 10:**
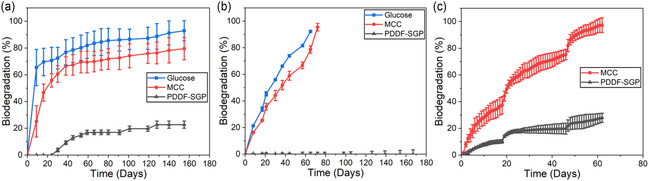
PDDF biodegradability tests against reference materials (glucose and/or micro‐crystalline cellulose (MCC)). a) Freshwater biodegradation profile. b) Soil biodegradation profile. c) Compost biodegradation profile.

To explore another EoL option for PDDF, biodegradation of PDDF‐SGP was conducted in freshwater, soil, and compost environments, and the results are summarized in **Figure** **10**. The test is considered valid if the degree of the biodegradation of the reference material (glucose or microcrystalline cellulose (MCC)) is more than 60% at the end of the test for the freshwater environment, more than 70% in 6 months for the soil environment, and 70% after a specific timeframe (usually around 45 days) for compost environment. The results for PBAT were reported previously by Zheng *et al.* ^[^ ^62^ ^]^ In the freshwater environment, PDDF‐SGP achieved ≈ 17% biodegradation in 72 days, while PBAT reached around 35% within the same period. Therefore, the estimated EoL time, based on first‐order kinetics, is 395 days for PBAT, whereas for PDDF‐SGP, it is projected to be 990 days under the same conditions. In the soil environment, PBAT reached around 20% biodegradation in 96 days but for PDDF no measurable biodegradation was observed after 96 days. However, in the compost environment, PDDF‐SGP reached about 28% biodegradation in 62 days, with an estimated EoL time of 475 days based on first‐order kinetics. The slower biodegradation rate of PDDF‐SGP compared to PBAT may be attributed to two key factors. First, PDDF‐SGP has a higher degree of crystallinity, which makes the polymer structure more rigid and less accessible to microbial attack.^[^ ^63^ ^]^ Second, the incorporation of a long‐chain alkyl in PDDF‐SGP results in fewer ester groups per unit length of the polymer chain. Since ester groups are the primary sites for hydrolytic degradation, their lower density in PDDF likely reduces its susceptibility to biodegradation.^[^ ^64^ ^]^

## 3 Conclusions

Both SGP and ROP routes were utilized to synthesize biobased polyester PDDF with low to high molecular weights (*M*_w_ up to 1.0 MDa). Compared to PBAT, both PDDF and PBAT are thermally stable up to 350 °C and have a similar *T*_m_, while PDDF exhibits a higher degree of crystallinity and a higher lamellar thickness. Moreover, the increase in percent crystallinity contributes to an increase in Young's modulus (by ≈3 ×) and a decrease in elongation at break for PDDF compared to PBAT. Both PDDF‐SGP and PDDF‐ROP exhibit shear‐thinning behavior at high frequencies; however, for PDDF‐ROP the Newtonian plateau couldn't be observed because its melt viscosity is very high (due to its very high molecular weight) and the lowest frequency testable was limited. PDDF‐SGP and PBAT melts have about the same elongation viscosities at low times, and both show strain hardening in elongation. The rate of biodegradation of PDDF was about half that for PBAT in freshwater, and PDDF is also partially biodegradable in compost environment but not in the type of soil tested here.

Notably, PDDF exhibits superior gas (oxygen, water vapor, and carbon dioxide) barrier properties to PBAT and also higher barriers toward oxygen and carbon dioxide permeation than LLDPE. Furthermore, PDDF demonstrates excellent chemical circularity, through dual closed‐loop circular cycles, either by base‐catalyzed depolymerization to cyclic monomer DDFL, followed by repolymerization via the ROP route, or by simple methanolysis to diester and diol monomers, followed by repolymerization via the SGP route. Overall, these superior barrier properties that are attributed to the inherently rigid, polar, H‐bonding furan rings and the long hydrophobic dodecylene segments, coupled with partial biodegradation and closed‐loop circularity, showcase the potential of the biobased PDDF as a more sustainable alternative for packaging.

## 4. Experimental Section

1

1.1

##### Materials

All synthesis and manipulations requiring dry inert atmosphere were performed under an argon/nitrogen atmosphere using standard Schlenk techniques or in a glovebox. HPLC‐grade organic solvents were first sparged extensively with nitrogen during filling 20 L solvent reservoirs and then dried by passage through activated alumina (for dichloromethane, DCM) followed by passage through Q‐5 supported copper catalyst (for toluene and hexanes) stainless‐steel columns. Benzyl alcohol (BnOH) was purchased from Alfa Aesar Chemical Co., purified by distillation over CaH_2_, and stored over activated Davison 4 Å molecular sieves. Dimethyl Furan‐2,5‐dicarboxylate, 2,5‐furandicarboxylic acid (FDCA, Accela), 1,12‐dodecanediol (DD), Tris[*N,N*‐bis(trimethylsilyl)amide]lanthanum(III) (La(NTMS)_3_), Phosphazene base ^*t*^Bu‐P_4_ solution (0.8M in hexane), 1,8‐diazabicyclo 5.4.0 undec‐7‐ene (DBU), Tin(II) 2‐ethylhexanoate (Sn(Oct)_2_), were purchased from Sigma‐Aldrich Chemical Co. and used as received. Titanium (IV) isopropoxide was purchased from Acros Organics. NMR solvents (CDCl_3_, D_2_O) were used as received. For comparison, film‐grade LLDPE (LyondellBasell Petrothene® NA963083) with a melt‐flow index of 0.7 g 10 min^−1^ and PBAT (BASF Ecoflex® C1200) were also tested. LLDPE was generously supplied by Amcor plc, while PBAT was sourced from Azelis Americas.

##### Instruments and Characterizations

*Nuclear magnetic resonance (NMR) analysis:* All NMR spectra were recorded on a Varian Inova or Bruker AV‐III 400 MHz spectrometer (400 MHz, ^1^ H; 100 MHz, ^13^C). Chemical shifts for ^1^ H and ^13^C spectra were referenced to internal solvent resonances and are reported as parts per million relative to CHCl_3_.

*Single crystal X‐ray diffraction:* Single crystal X‐ray diffraction patterns were collected on a Bruker SMART APEX CCD Diffractometer using Mo Kα (*λ* = 0.71073 Å) radiation at 100 K. The structures were solved by direct methods and refined using the Bruker SHELXTL program library by full‐matrix least squares on square modulus of structure factors |F|^2^ of all reflections. All non‐hydrogen atoms were refined with anisotropic displacement parameters, whereas hydrogen atoms were included in the structure factor calculations at idealized positions. Crystallographic data for the structure of DDFL (CCDC‐2 359 161) has been deposited with the Cambridge Crystallographic Data Center as supplementary publications. These data can be obtained free of charge from The Cambridge Crystallographic Data Centre via www.ccdc.cam.ac.UK/data_request/cif.

*Wide angle X‐Ray scattering (WAXS) and small‐angle X‐ray scattering (SAXS):* WAXS and SAXS measurements were performed using a Xenocs XEUSS 3.0 with Cu‐Kα radiation. The microfocus source was operated at 50 kV and 0.6 mA and a q‐range of 0.15 to 3.4 Å^−1^ and 0.01 to 0.3 Å^−1^ with an exposure time of 60 and 120 mins was used for WAXS and SAXS, respectively. Fractional crystallinity was calculated using a program that fits a baseline followed by a Gaussian curve to each peak and the amorphous halo.

*Absolute molecular weight measurements:* Measurements of polymer absolute weight‐average molecular weight (*M*_w_), number‐average molecular weight (*M*_n_), and molecular weight dispersity (*Đ *= *M*_w_ *M*_n_^−1^) were performed via size‐exclusion chromatography (SEC). The SEC instrument consisted of an Agilent HPLC system equipped with one guard column and three PLgel 5 μm mixed‐C gel permeation columns unless indicated otherwise and coupled with a Wyatt DAWN HELEOS II multi (18)‐angle light scattering detector and a Wyatt Optilab TrEX dRI detector; the analysis was performed at 40 °C using chloroform as the eluent at a flow rate of 1.0 mL min^−1^, using Wyatt ASTRA 7.1.2 molecular weight characterization software. The refractive index increments (d*n* d*c* ^−1^) of PDDF polymers were determined to be 0.0328 ± 0.0007 mL g^−1^ and 0.0308 ± 0.0008 mL g^−1^, respectively, obtained by batch experiments using Wyatt Optilab TrEX dRI detector and calculated using ASTRA software. Polymer solutions were prepared in chloroform and injected into dRI detector by Harvard Apparatus pump 11 at a flow rate of 0.2 mL min^−1^. A series of known concentrations were injected and the change in refractive index was measured to obtain a plot of change in refractive index versus change in concentration ranging from 0.5 to 10.0 mg mL^−1^.

*Thermal analysis:* Melting transition (*T*_m_) and glass transition (*T*_g_) temperatures were measured by DSC on a TA Instruments DSC 2500. Indium metal, tin, and biphenyl were used for calibration. A sample was cooled to −85 °C and then heated to 180 °C with a heating rate of 10 °C min^−1^ (first heating). The sample was held at 180 °C for 5 min to erase the thermal history and then cooled down to −85°C at a cooling rate of 10°C min^−1^ (cooling) and finally heated again to 180°C at a heating rate of 10°C min^−1^ (second heating). In the case of isothermal crystallization, the samples were initially heated to 180°C, quenched at a rate of 80°C min^−1^ to the required crystallization temperature, and held for 30 mins to reach maximum crystallinity. Aluminum pans were used with a sample size ranging from 5 to 15 mg. Thermal stability of the polymer using a TA Instruments TGA55. Polymer samples were heated from 40°C to 700°C at a heating rate of 10°C min^−1^ in nitrogen gas. 100 μL platinum pans were used with a sample size varied between 10 and 20 mg. Values of *T*_max_ were obtained from derivative (wt% °C^−1^) versus temperature (°C) plots.

Mechanical analysis: Tensile stress/strain testing was performed by a 2000 lb. SSTM tester from United Testing Systems (10 kN load cell) on dog‐bone‐shaped test specimens (ASTM D61708 standard) prepared *via* compression molding using a Carver Bench Top Laboratory Press (Model 3925) equipped with a two‐column hydraulic unit (Carver, Model 3925, maximum force 24 000 psi) unless indicated otherwise. Isolated polymer materials were loaded between non‐stick polyimide sheets into a stainless‐steel mold with inset dimensions 92 × 62 × 0.38 mm fabricated inhouse and compressed between two 6'' × 6'' steel electrically heated platens (EHP) clamp force 10 000 psi, at temperature of 180 °C. Specimens for analysis were generated *via* compression molding and cut using an ASTM D1708 cutting die to standard dimensions. Mechanical behavior was averaged for all the specimens measured for each individual species investigated. Thickness (0.38 ± 0.01 mm), width (5.0 mm), and length (22.3 ± 0.2 mm) of the dog‐bone specimens were measured for normalization of data. Test specimens were affixed into the screw‐tight grip frame. Tensile stress and strain were measured to the point of material break at a grip extension speed of 0.5 in min^−1^ at ambient conditions.

*Rheology analysis:* Rheological properties were investigated using a TA instrument, ARES G2 constant strain rheometer. 25 mm parallel stainless‐steel plates and a 1 mm gap were used to measure the storage modulus, loss modulus, and complex viscosity. A constant temperature of 190 °C and a frequency range from 600 rad s^−1^ to 0.01 rad s^−1^ were chosen for the shear viscosity. This temperature was selected to replicate the die temperature for film‐blowing of LLDPE. Extensional viscosity was measured using the extensional viscosity fixture of the ARES G2 rheometer at 130 °C at an extensional rate of 0.3 s^−1^. The former value was selected to be slightly higher than the melting temperature to prevent sagging. The latter value was chosen because a Hencky strain of 2.5 at an elongational rate of 0.3 s^−1^ was found to be a representative value for film blowing. Dynamic mechanical analysis (DMA) for the polymers was performed using a linear tension fixture geometry on the ARES G2. The test was conducted at a temperature range of −70 °C to the sample melting temperature, a frequency of 1 Hz, and a temperature step of 3 °C.

*Permeability measurements:* CO_2_ and O_2_ permeability of the films was measured at 1 bar and 25 °C in a constant volume, variable pressure barometric device with a 47 mm HP filter holder. Prior to the experiment, the system was left under vacuum for 24 h to remove any humidity and previously absorbed gases. Upstream pressure was monitored using a PX‐409‐USHB pressure transducer with a full‐scale of 1000 psi while the downstream pressure was monitored using another PX‐409‐USHB with a full scale of 260 torr. The change in downstream pressure over time was recorded and the permeability (Ρ) at steady state can be calculated using Equation. (6)

(6) $$\text{Ρ}=\frac{lV_{down}}{ART\Delta p}.{\frac{dp}{dt}}_{down}$$

where, *l* and *A* are the thickness and the area of the film, respectively, *R* is the universal gas constant, *T* is the absolute temperature, $V_{down}$ is the downstream volume, Δp is the pressure difference across the film, and ${\frac{dp}{dt}}_{down}$ is the steady‐state slope of pressure versus time in the downstream. CO_2_ and O_2_ permeability values were normalized to film thickness.

WVTR was measured according to ASTM E96 (dry cup method) by first fixing a dry sample (of mass m_0_) on a vail filled with desiccant (CaSO_4_) in a desiccator saturated with water vapor and then monitoring the increase in mass (m_g_) as a function of exposure time. The vials covered with the film were weighed at certain times until 21 days. Weight changes of the vial were plotted versus times and then slopes of the plot of each sample were obtained. The thickness and area of each sample were measured prior to testing. WVTR was calculated using Equation (7)

(7) $$WVTR={\frac{dm_{g}}{dt}|}_{s.s}.\frac{l}{A}$$

where, ${\frac{dm_{g}}{dt}|}_{s.s}$is the steady‐state slope of mass gained versus time and *l* and *A* are the thickness and the area of the film, respectively. WVTR values were normalized to film thickness.

*Biodegradability measurements:* Freshwater biodegradability study of the polyesters followed the guidelines of ISO 14 851. Each polyester underwen*t* testing in triplicate using 300 mL biological oxygen demand (BOD) glass bottles from VWR International. In each bottle, 200 mL of an aqueous medium containing KH_2_PO_4_ (85 mg L^−1^), K_2_HPO_4_ (217.5 mg L^−1^), Na_2_HPO_4_ (334 mg L^−1^), NH_4_Cl (15 mg L^−1^), MgSO_4_·7H_2_O (22.5 mg L^−1^), CaCl_2_·2H_2_O (36.4 mg L^−1^), and FeCl_3_·6H_2_O (0.25 mg L^−1^) from Fisher Scientific was mixed with activated sludge obtained from a wastewater treatment plant in Lemont, IL, resulting in a total solid concentration of 60 mg L^−1^ in the BOD bottle. Polyester samples (PDDF or PBAT) in film form (2 mm × 2 mm × 0.5 mm) were added to each BOD bottle, with a total organic carbon content of 9.0 mg. Triplicate sets of negative controls (without additional carbon) and positive controls (with 9.0 mg total organic carbon from d‐glucose and cellulose) were also prepared. Incubation of all bottles occurred in a New Brunswick Scientific incubator shaker (Eppendorf, model I‐24) at 25 °C and 150 rpm. Oxygen consumption, indicative of BOD, was measured using a pH/RDO/DO meter (Thermo Fisher Scientific, model Orion Star A216) according to ISO 14 851 standards as well. The biodegradability percentage was calculated using Equation (8).

(8) $$Biodegradation\;\%=\frac{BOD_{sample}-BOD_{blank}}{C\times Th_{OD}}$$

BOD_sample_ and BOD_blank_ represent the recorded measurements from the sample and blank bioreactor, respectively. C denotes the amount of sample added, while Th_OD_ stands for the theoretical oxygen demand value of the sample, derived from its chemical formula under the assumption of full oxidation to CO_2_ and H_2_O. The procedure mandates that the positive control (glucose and microcrystalline cellulose) achieve a 60% biodegradation level by the test's conclusion, with the standard deviation of each sample being under 20% of the mean.

Biodegradability tests in the soil environment (soil respiration tests) were conducted according to ASTM D5988‐18. The soil respiration tests were set up in 1 L Kimble bottles (with Wheaton Black Phenolic Cap and Gray Butyl Septa and Flange, DWK Life Sciences) in triplicate. Each test bottle contained 150 g of soil (a mixture of soil samples taken from local forests in Illinois). To each test bottle, additional water was added to reach 70% of the field capacity (water holding capacity) of the soil sample. The field capacity of the soil was measured using the ASTM D2980‐17E01. Each of the polymer sample (10 mm × 10 mm × 0.5 mm size) containing 0.30 g of total organic carbon was added to the BOD bottles and well mixed with the soil. A negative control with no additional content and two positive controls with glucose and cellulose containing 0.30 g total organic carbon were also set up in triplicate. The degradation of the polymers, glucose, and starch was monitored by measuring the CO_2_ volume released into the headspace of the bottles. Samples collected from the headspace were analyzed by gas chromatography (Shimadzu, model GC‐2014). The biodegradation of polymers in percentage is calculated as shown in equation (9)

(9) $$Biodegradation\;\%=\frac{V_{CO_{2},sample}-V_{CO_{2},Blank}}{ThV_{CO_{2},sample}}$$

The ${\text{V}}_{{\text{CO}}_{2},\text{sample}}$ is the theoretical production volume of CO_2_ calculated from the carbon mass in polymer samples.

End‐of‐life tests in industrial composting environments were conducted according to ASTM D5338‐15 method. The AER‐800 Research Respirometer (Challenge Technology, USA) was used to quantify the CO_2_ production rate in aerobic mode. The compost was acquired from a local compost facility (Land‐and‐Lakes Company, Romeoville, IL, USA) and screened through a 10 mm sieve. Microcrystalline cellulose (particle size 0.05 mm, Acros Organics) was used as positive control. Each chamber was loaded with compost of 40 g total solid and 1.5 g of microcrystalline cellulose or PDDF polymer film samples (cut to 10 mm × 10 mm × 0.5 mm pieces). Additionally, three chambers were set up only with compost as the negative control. Each positive control or polymer was also run in triplicate (three test chambers). Biodegradation test chambers were maintained in a 58 ± 2 °C water bath, with a built‐in KOH trap to absorb the produced CO_2_. The KOH solution (45% in water, Sigma‐Aldrich) was changed periodically. Continuous flow of ultrahigh purity O_2_ was maintained at 5 psi to compensate for the trapped CO_2_ and the flow rate of O_2_ into each chamber in mg was recorded by the respirometer as the CO_2_ production rate. Milli‐Q water was periodically added to each chamber to maintain the relative humidity at ≈55%. The percentage biodegradation of polymers was calculated using:

(10) $$\%\;Biodegradation=\frac{m_{CO_{2},sample}-m_{CO_{2},blank}}{Thm_{CO_{2},sample}}\times 100\%$$

${\text{Thm}}_{{CO}_{2},\text{sample}}$ was the theoretical production mass of CO_2_ calculated from the mass of carbon in polymer samples.

All samples were compression molded in a Carver laboratory press at 5 metric tons and a temperature of 180 °C to a thickness between 0.4 and 0.5 mm. Following compression molding, the mold was left to cool down slowly until it reached 40 °C. Immediately prior to measurement, samples were dried in a vacuum oven for 24 h at 80 °C to remove any residual moisture.

## Conflict of Interest

The authors declare no conflict of interest.

## Supporting information

Supplementary Material

## Data Availability

The data that support the findings of this study are available in the supplementary material of this article.
