# Supplementary material for: Biobased Poly(dodecylene Furanoate) with Inherent Advantages in Performance and Circularity
Source: ChemSusChem. 2025 Jul 10;18(17):e202501080. doi: 10.1002/cssc.202501080 (PMC12404017; doi:10.1002/cssc.202501080)
Supplement: Supplementary file 1 — Supplementary Material [file CSSC-18-e202501080-s001.pdf]

Supporting Information

Biobased Poly(dodecylene furanoate) with Inherent Advantages in Performance and Circularity

Hesham Aboukeila, Eswara Rao Chokkapu, Hang-Fei Tu, Onkar Singh, Wilfred T. Diment, Shu Xu, Meltem Urgun-Demirtas, John Klier, George W. Huber, Brian P. Grady, and Eugene Y.-X. Chen

Table of Contents

Supplementary Methods

General Polymerization Procedures 3

Ring-opening polymerization (ROP) 3

Step-growth polymerization (SGP) 3

Experimental Details 3

Sythesis of 1,12-dodecane 2,5-furanoate lactone (DDFL) 3

ROP of dodecane 2,5-furanoate lactone (DDFL) 6

PDDF by step-growth polymerization (SGP) 7

Chemical recyclability of poly(dodecane 2,5-furanoate) (PDDF) 7

Supplementary Figures

Figure S1. ^{1}H NMR ($CDCl_3$, 23 °C) spectrum of 2,5-furanoate lactone (DDFL) by Yamaguchi 4

Figure S2. ^{1}H NMR ($CDCl_3$, 23 °C) spectrum of 2,5-furanoate lactone (DDFL) 5

Figure S3. ^{13}C NMR ($CDCl_3$, 23 °C) spectrum of 2,5-furanoate lactone (DDFL) 6

Figure S4. ^{1}H ($CDCl_3$, 23 °C) spectrum of PDDF polymer by ROP using $La(NTMS)_3$ as the catalyst 7

Figure S5. ^{1}H ($CDCl_3$, 23 °C) spectrum of PDDF polymer by SGP using $Ti[OC(CH_3)_3]_4$ as the catalyst 7

Figure S6. ^{1}H (CDCl3, 23 °C) spectrum of dimethyl furan-2,5-dicarboxylate and DD mixture 9

Figure S7. Representative SEC trace of PDDF by ROP 9

Supplementary Methods

General Polymerization Procedures

Ring-opening polymerization (ROP)

The ROP reactions were performed inside a N_2-filled glovebox or on a Schlenk line in a sealed 10.0 mL glass reactors at a predetermined temperature. The reactor was charged with a specified amount of monomer, catalyst, and initiator (as detailed in the polymerization tables) inside the glovebox. The mixture, in a solvent, was stirred at ambient temperature (~25 °C) or at different temperatures, both with and without solvent (neat conditions). The polymerization was initiated by rapid addition of the catalyst to the monomer. After a desired time period, the polymerization was immediately quenched by addition of 1 (wt%) of HCl in chloroform (10 mg/mL) and a 0.02 mL of aliquot was taken from the reaction mixture and prepared for ^{1}H NMR analysis to obtain the percent monomer conversion data. The quenched mixture was then precipitated into 50 mL of methanol while stirring, filtered, washed with methanol to remove any unreacted monomer, and dried in a vacuum oven at 60 °C for 24 h to a constant weight.

Step-growth polymerization (SGP)

Synthesis of PDDF by the SGP route was performed following a two-stage polycondensation method. In the first step (esterification), oligomers were synthesized without the application of vacuum on the reaction mixture by heating it to 150 °C for 15 h until the theoretical amount of methanol was collected in the side arm of the condenser apparatus. Then in a second step (polycondensation), the condenser apparatus was removed, and a vacuum (50 mTorr) was applied slowly over a period of about 30 min to minimize the sublimation of oligomers. The PDDF was synthesized at 220 °C and pressure 50 mTorr for 15 h.

Experimental Details

Synthesis of 1,12-dodecane 2,5-furanoate lactone (DDFL)

Method 1: Yamaguchi esterification

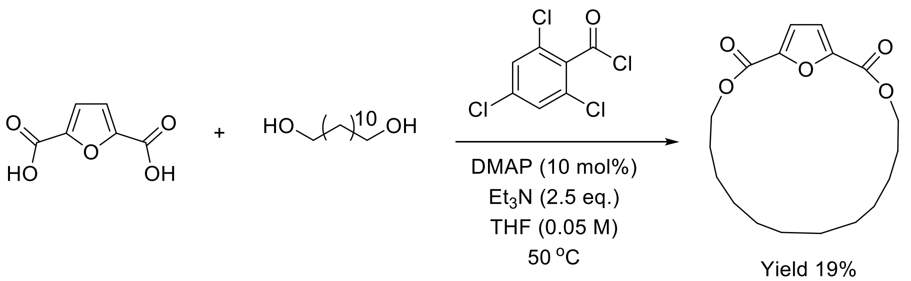

Scheme S1: Synthesis of dodecane 2,5-furanoate lactone (DDFL) by Yamaguchi esterification

The Yamaguchi esterification began by dissolving FDCA (1.0 g, 6.41 mmol) in THF in a dry round-bottom flask under an inert atmosphere. To this solution, 2,4,6-trichlorobenzoyl chloride (the Yamaguchi reagent) (3.11 g, 12.8 mmol) was added, followed by the addition of 2.5 equiv. Et_3N to neutralize the hydrogen chloride generated during the reaction. The mixture was stirred at a slightly elevated temperature (around 40–50 °C) to form the mixed anhydride. Once the formation of the mixed anhydride was confirmed using

thin-layer chromatography (TLC), the alcohol DD was added along with a catalytic amount of 4-dimethylaminopyridine (DMAP, 10 mol%). The reaction mixture was then stirred at slightly elevated temperatures (50–60 °C) for 6 h to produce the desired product. After removal of the solvent under reduced pressure, the residue was washed with brine solution and extracted with dichloromethane. The extracted organic layer was dried over anhydrous Na_2SO_4, and the solvent was removed. The resulting mixture was purified by column chromatography (ethyl acetate, 15%), yielding 19% of DDFL. ^{1}H NMR (400 MHz, $CDCl_3$, ppm) δ 7.29 (s, 2H), 4.37 (t, 4H), 1.75 (m, 4H), 1.53 (m, 4H) 1.40-1.35 (m, 13H).

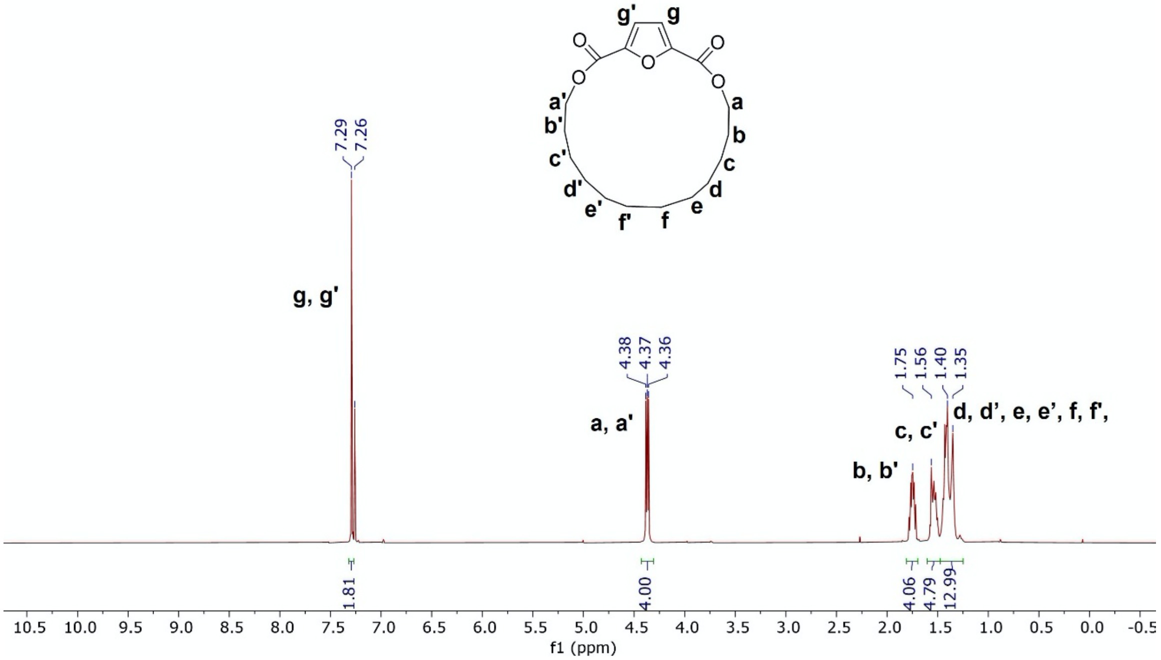

Figure S1. ^{1}H NMR ($CDCl_3$, 23 °C) spectrum of 2,5-furanoate lactone (DDFL) by Yamaguchi esterification.

Method 2: acid chloride DFCA-Cl_2

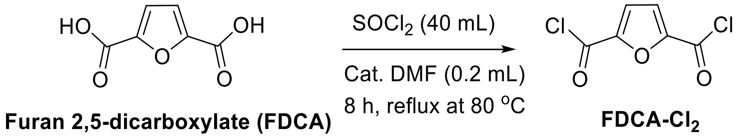

Scheme S2: Synthesis of FDCA-Cl_2 precursor

FDCA-Cl_2 was prepared by reacting FDCA (20 g, 0.128 mol) with $SOCl_2$ (40 mL, 0.336 mol) and catalytic amount of DMF (0.2 mL, 0.0026 mol) under reflux at 80 °C for 8 h.

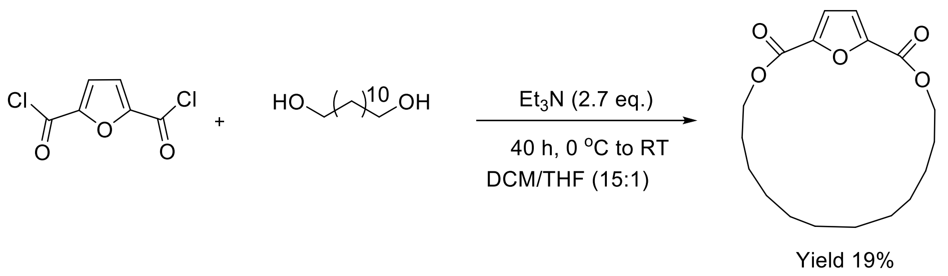

Scheme S3: Synthesis of dodecane 2,5-furanoate lactone (DDFL)

A round-bottom flask was charged with 150 mL of dichloromethane and cooled to 0 °C. Under stirring, 64.8 mmol (6.50 g) of Et_3N were added. Then, 27.0 mmol (5.49 g) of 1,12-dodecanediol in 10 mL of THF were introduced into the reaction solution. Subsequently, 25.9 mmol (5.00 g) of FDCA-Cl_2 in 100 mL of DCM were added dropwise to the reaction mixture using a pressure-equalizing funnel at 0 °C. After stirring for 48 h at room temperature, the solvent was removed under reduced pressure. The mixture was washed with brine solution and extracted with dichloromethane. The organic layer was dried over anhydrous Na_2SO_4 and evaporated. The reaction mixture was purified by column chromatography (ethyl acetate, 15%), yielding 19% of DDFL.

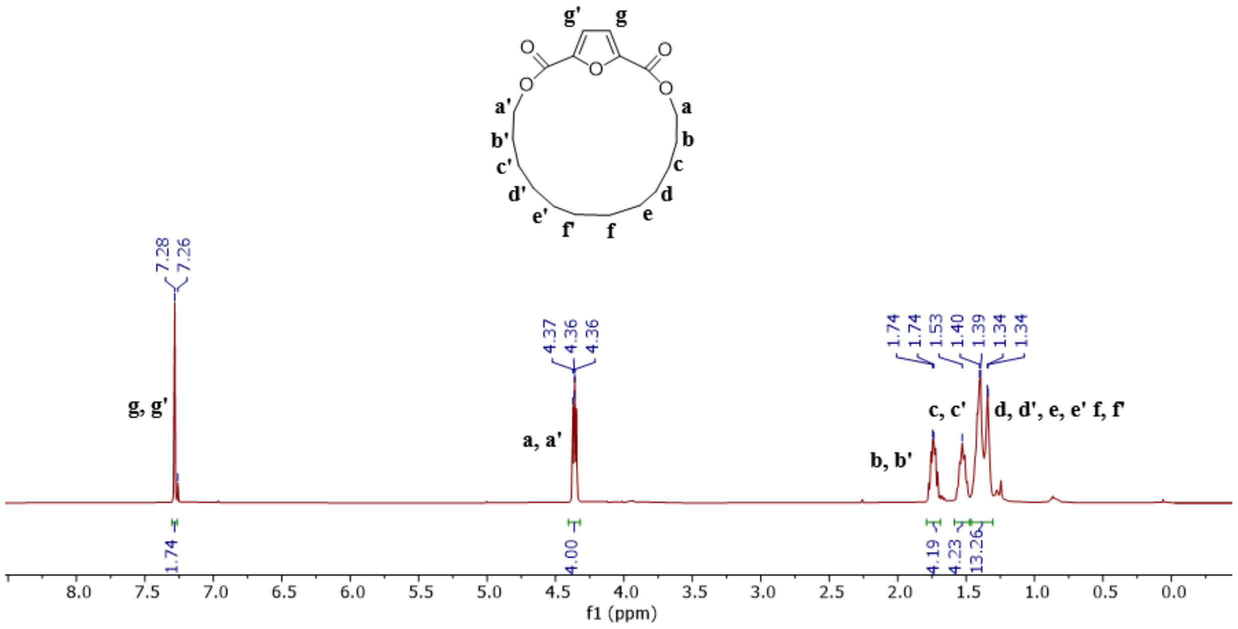

Figure S2. ^{1}H NMR ($CDCl_3$, 23 °C) spectrum of 2,5-furanoate lactone (DDFL).

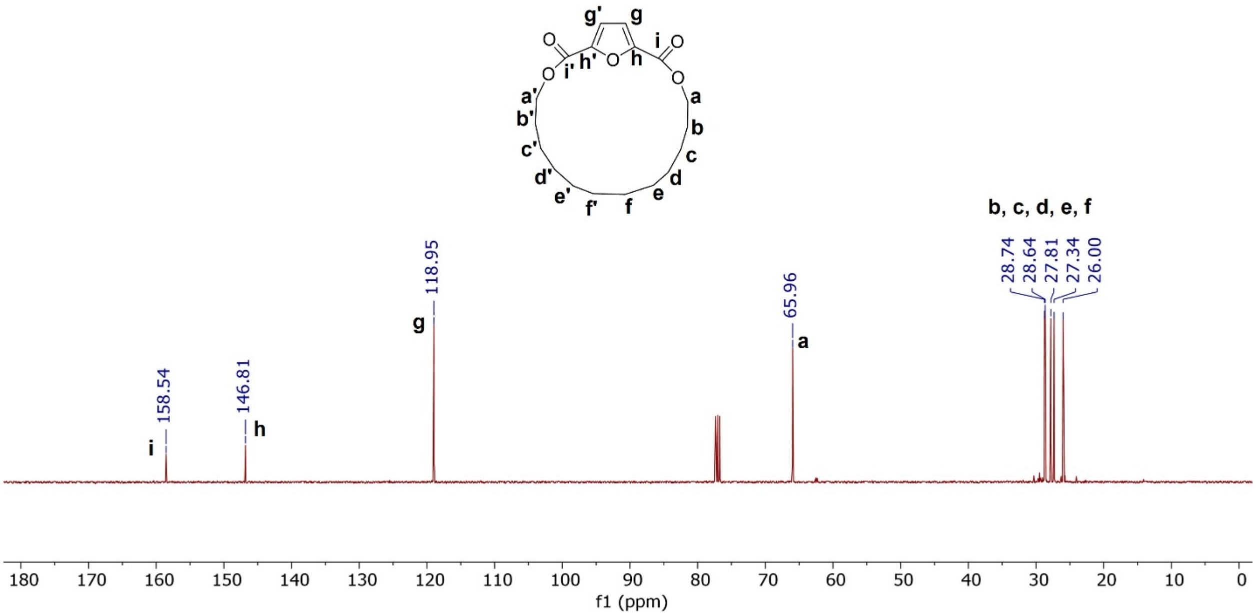

Figure S3. ^{13}C NMR ($CDCl_3$, 23 °C) spectrum of 2,5-furanoate lactone (DDFL).

ROP of dodecane 2,5-furanoate lactone (DDFL)

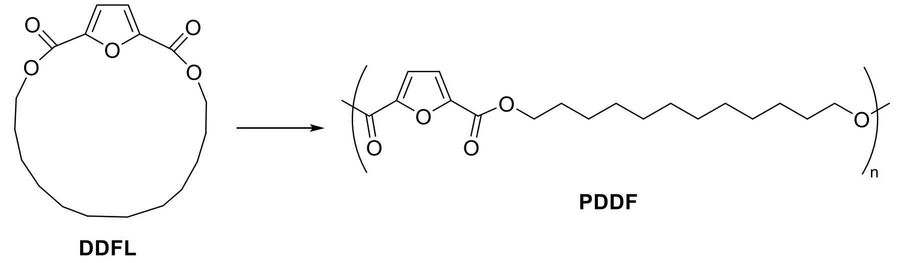

Scheme S4: Synthesis of poly(dodecane 2,5-furanoate) by ROP

Polymerization was conducted in a small, oven-dried Schenk tube. Inside a glovebox, the reactor was charged with specified amounts of monomer, catalyst, and initiator as detailed in the polymerization tables. The mixture was stirred in a solvent at ambient temperature (~25 °C) as well as under different temperature conditions, both with and without solvent (neat conditions). After the reaction, the mixture was quenched with acidic CH_2Cl_2 and precipitated in methanol. The product was analyzed by ^1H-NMR to determine the percent monomer conversion. Using $La(NTMS)_3$ as the catalyst, the monomer conversion reached 100%, and the isolated yield of PDDF was 95.0%. ^1H NMR (400 MHz, $CDCl_3$, ppm) δ 7.18 (s, 2H), 4.32 (t, 4H), 1.75 (m, 4H), 1.39-1.27 (m, 17H).

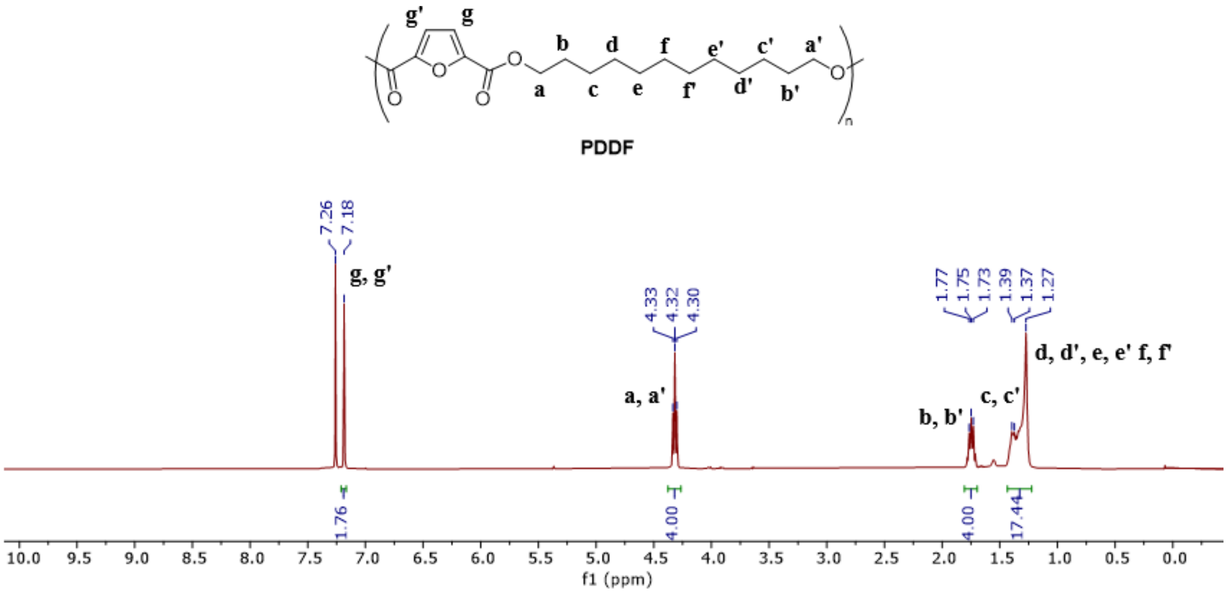

Figure S4. ^{1}H NMR ($CDCl_3$, 23 °C) spectrum of poly(dodecane 2,5-furanoate) (PDDF) by ROP using $La(NTMS)_3$ as the catalyst.

PDDF by step-growth polymerization (SGP)

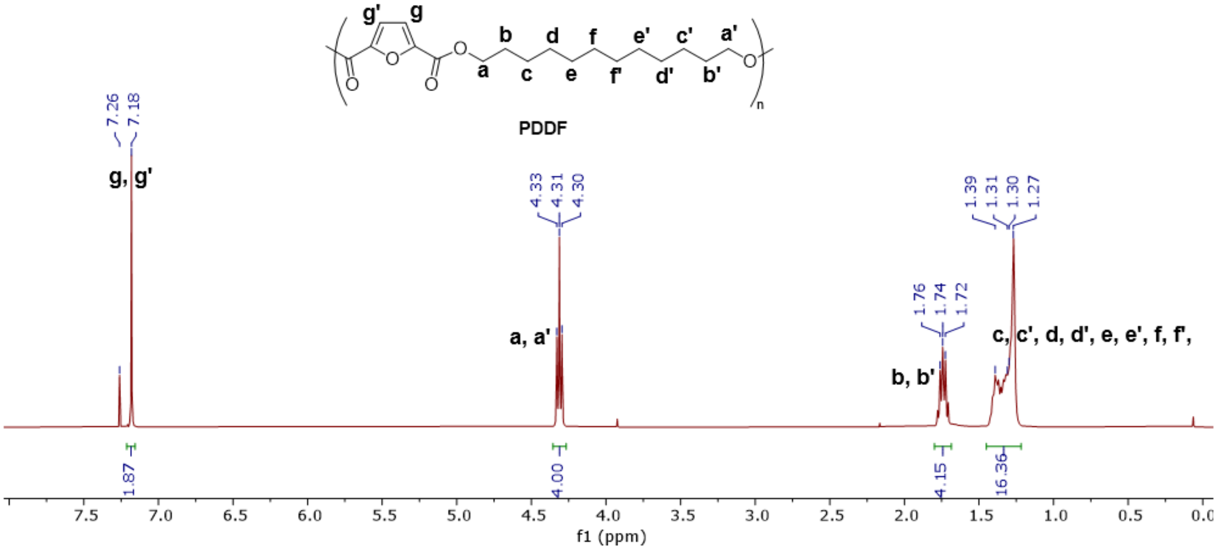

Figure S5. ^{1}H NMR ($CDCl_3$, 23 °C) spectrum of poly(dodecane 2,5-furanoate) (PDDF) by SGP using $Ti[OC(CH_3)_3]_4$ as the catalyst.

Chemical recyclability of poly(dodecane 2,5-furanoate) (PDDF)

Catalyzed closed loop:

In this study, 1.9 g of PDDF was chemically recycled to obtain cyclic oligomers. The recycling process employed phosphazene base ^{t}Bu-P_4 (1.5 mol%) at 120 °C under vacuum for 15 h. Depolymerization was carried out in a sublimator, and the cyclic oligomers recovered in the cold finger with a yield of 71.0%.

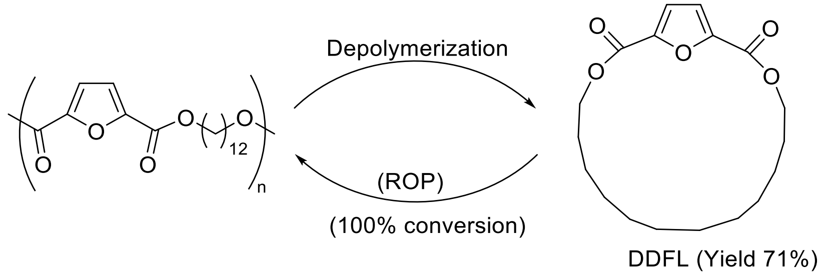

Scheme S6: Depolymerization of poly(dodecane 2,5-furanoate) by P_4-*t*-Bu.

Methanolysis closed loop:

In this study, 0.5 g of PDDF polymer was placed in a 20.0 mL glass autoclave along with 5.0 mL of methanol. To this, 30 wt% Et_3N was added. The reactor was sealed, and the reaction was carried out at 120 °C for 18 h. After the reaction, the mixture was allowed to cool to room temperature, and methanol and Et_3N were removed using a rotary evaporator. The product was analyzed by ^{1}H NMR, confirming quantitative conversion.

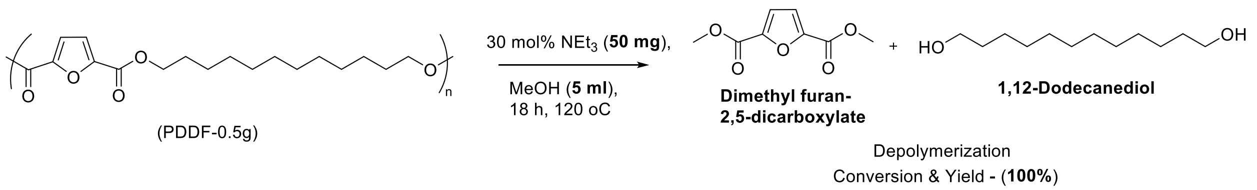

Scheme S7: Depolymerization of poly(dodecane 2,5-furanoate) by Et_3N

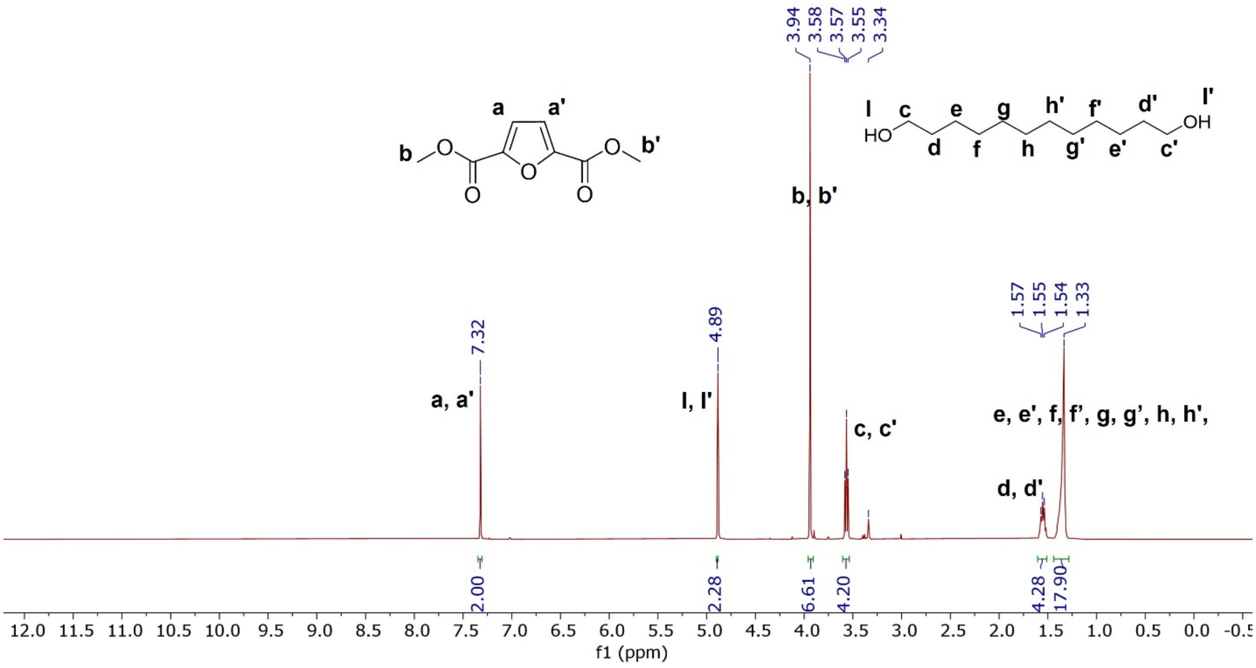

Figure S6. ^{1}H NMR ($CDCl_3$, 23 °C) spectrum of dimethyl furan-2,5-dicarboxylate and 1,12-DD monomers mixture.

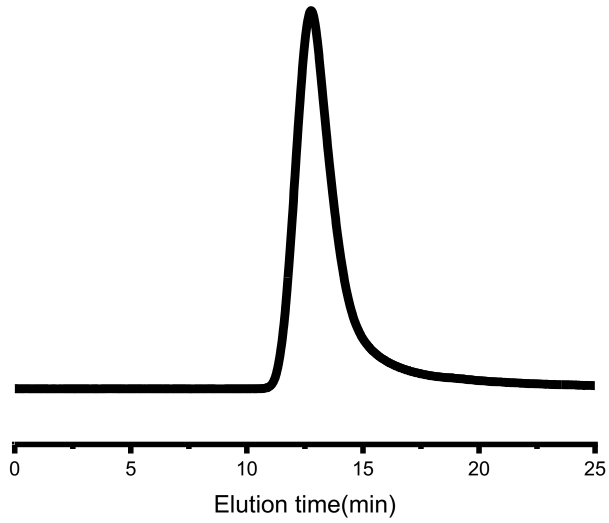

Figure S7. Representative SEC trace of PDDF (M_n = 807 kDa, $Đ$ = 1.41, by ROP).
